# Peripheral Blood Transcripts Predict Preoperative Obstructive Total Anomalous Pulmonary Venous Connection

**DOI:** 10.3389/fcvm.2022.892000

**Published:** 2022-05-31

**Authors:** Zunmin Wan, Xiaohong Li, Jinghua Sun, Xiaohua Li, Zhongzhen Liu, Haojian Dong, Qing Zhou, Hailong Qiu, Jinjin Xu, Tingyu Yang, Wen-Jing Wang, Yanqiu Ou

**Affiliations:** ^1^College of Life Sciences, University of Chinese Academy of Sciences, Beijing, China; ^2^Guangdong Cardiovascular Institute, Guangdong Provincial People's Hospital, Guangdong Academy of Medical Sciences, Guangzhou, China; ^3^BGI-Shenzhen, Shenzhen, China

**Keywords:** total anomalous pulmonary venous connection, preoperative obstruction, machine learning, peripheral leukocyte transcripts, MEG3

## Abstract

The lack of accessible noninvasive tools to examine the molecular alterations limits our understanding of the causes of total anomalous pulmonary venous connection (TAPVC), as well as the identification of effective operational strategies. Here, we consecutively enrolled peripheral leukocyte transcripts of 26 preoperative obstructive and 22 non-obstructive patients with TAPVC. Two-hundred and fifty six differentially expressed mRNA and 27 differentially expressed long noncoding RNA transcripts were dysregulated. The up-regulated mRNA was enriched in the hydrogen peroxide catabolic process, response to mechanical stimulus, neutrophil degranulation, hemostasis, response to bacterium, and the NABA CORE MATRISOME pathway, all of which are associated with the development of fibrosis. Furthermore, we constructed predictive models using multiple machine-learning algorithms and tested the performance in the validation set. The mRNA *NR3C2* and lncRNA *MEG3* were screened based on multiple iterations. The random forest prediction model can predict preoperative obstruction patients in the validation set with high accuracy (area under curve = 1; sensitivity = 1). These data highlight the potential of peripheral leukocyte transcripts to evaluate obstructive-related pathophysiological alterations, leading to precision healthcare solutions that could improve patient survival after surgery. It also provides a novel direction for the study of preoperative obstructive TAPVC.

## Introduction

Total anomalous pulmonary venous connection (TAPVC) is one of the most critical congenital heart diseases (CHD), accounting for 1 to 3% of all CHD. All four pulmonary venous of TAPVC patients cannot be properly connected to the left atrium, and abnormal connections to the right atrium or the systemic venous system may occur (1). Patients with TAPVC have a mortality rate of up to 80% in the absence of surgical intervention (2). However, postoperative pulmonary venous obstruction (PVO) occurs in 5–20% of patients resulting in increased mortality and morbidity which poses an ongoing surgical challenge (3–5).

Preoperative obstruction is a vital risk factor for postoperative obstruction, and 50% of patients with preoperative obstruction develop postoperative obstruction (6). Previous studies showed that, for preoperative obstructive patients, the use of sutureless technology can reduce the probability of postoperative PVO by avoiding pulmonary vein endothelial damage, preventing reactive intimal hyperplasia, and avoiding twisting and rotation (6, 7). In addition, some patients with primary preoperative obstruction may need heart–lung transplantation instead of repair surgery, because the existing surgical methods not only bring no benefits but will increase the pain and economic burden of multiple operations for children (8). Therefore, we need to identify preoperative obstruction to facilitate decision-making and operation processing to reduce the incidence of postoperative obstruction and improve surgical survival.

The following four conditions are currently used to confirm the diagnosis of preoperative obstruction: oxygen saturation, echocardiography data which indicated the existence of continuous pulmonary venous flow velocity >1.8 m/s, morphological features investigated with computed tomography angiography (CTA), and intraoperative assessment (9). Preoperative clarification of obstruction is more relevant for the prevention of postoperative obstruction, and CTA has limitations in its ability to detect intravenous or small venous obstruction in TAPVC and requires a high level of operator proficiency (6). Therefore, the development of an accurate and noninvasive predictive model to complement existing strategies is essential for the early prediction of preoperative obstruction. Some studies have constructed models to predict preoperative obstruction based on CTA images or pulmonary vein coefficients of variation (10, 11). However, these prediction models are not effective due to their still depend on image capture and the heterogeneity and complexity of the disease (11). Moreover, some patients may not be identified with a preoperative obstruction or have preoperative obstructive symptoms before surgery, but they already have the hypoplastic pulmonary venous which develops into progressive obstruction under the stimulation of the operation, the full length from the anastomosis to the pulmonary vein and leading to fetal morbidity (12). Therefore, we need features that can describe molecular alterations and predict preoperative obstruction independently of CTA images.

The location of the preoperative obstructive TAPVC lesion occurs in the pulmonary veins, and the presence of myofibroblasts is initially observed at autopsy (13). Further studies revealed an overgrowth of connective tissue with medial hypertrophy and intimal fibrosis, leading to extracellular matrix (ECM) deposition and subsequent obstruction (14). Therefore it can be assumed that venous vascular fibrosis leads to preoperative obstructive TAPVC. Fibrosis can affect any organ, of particular concern are the lungs, the cardiovascular system, the liver, etc (15). Leukocytes play a role in fibrosis, where stimulated tissue releases inflammatory mediators, which trigger an influx of leukocytes to the site of injury, and the activated leukocytes then further regulate pathological fibrosis (16). For example, identified alveolar macrophages specific to pulmonary fibrosis in single-cell analysis (17). Granulocytes [mast cells (18), eosinophils (19), neutrophils (20)] can partially influence fibrosis by secreting fibrogenic mediators and regulating the expression of matrixes (16); Macrophages and dendritic cells have different regulatory effects on the outcomes of fibrosis (21, 22). In addition to this, markers of elevated lipid values and systemic inflammation can be detected in the circulating blood of patients with atherosclerosis (23). Thus, blood cells circulating throughout the body are an important direction for liquid biopsies that can noninvasively characterize the molecular alterations and independent of CT images, which are ideal tissues for preoperative obstructive TAPVC. Therefore, we hypothesized that leukocyte transcripts can provide biomarkers for the prediction of preoperative obstructive TAPVC. In the current study, to test this hypothesis, we consecutively enrolled 26 preoperative obstructive and 22 non-obstructive TAPVC patients, performed and analyzed transcript profiling of their preoperative peripheral leukocytes. We observed differentially expressed mRNA and long noncoding RNA transcripts in obstruction vs. non-obstruction, the up-regulated mRNA in obstructive TAPVC were enriched in hydrogen peroxide catabolic process, response to mechanical stimulus, neutrophil degranulation, hemostasis, response to bacterium and NABA CORE MATRISOME pathways, all of them are associated with the development of fibrosis. Leukocyte subtype analysis revealed a low percentage of T-cells CD8 in preoperative obstructed TAPVC. Furthermore, *NR3C2* and *MEG3* were selected as molecular features and RF preoperative prediction model was constructed with an AUC of 0.95 in the training set and AUC of one in the validation set. To further verify the usefulness of the features, both GLM and support vector machine (SVM) prediction models were constructed, and the area under curve in the validation set both were one. We confirmed the hypothesis that leukocyte transcripts can evaluate obstructive-related pathophysiological alterations, providing a novel direction for the study of preoperative obstructive TAPVC.

## Methods

### Design and Population

This is a retrospective cohort study reported in a specialized cardiovascular institute in Guangzhou, China. We consecutively recruited 48 samples with TAPVC receiving repair operations during August 2016 and December 2019. Patients with functionally univentricular circulations were excluded. All patients received echocardiographic examination and CTA before surgery (Supplementary Figure 1).

As part of institutional standard procedures, all enrolled patients who were discharged alive from the hospital were required to return for outpatient follow-up visits at 1, 6, and 12 months after the initial operation, and then annually. For those patients who failed to come back for outpatient visits, trained doctor's assistants call for a telephone interview.

All procedures were performed according to the ethical standards of the Guangdong Provincial People's Hospital Human Subjects Committee (No. GDREC2018363H) and with the 1964 Helsinki Declaration and its later amendments or comparable ethical standards. Written informed consent was obtained from all participants or their guardians before study enrollment.

### Clinical Data Source

Clinical data were collected from the hospital CHD specialized database and extracted from the medical records and the outpatient records at the last available follow-up.

### Diagnosis of Preoperative Obstructive TAPVC

As reported previously, the diagnosis of preoperative obstruction was made by a combined evaluation of oxygen saturation, echocardiography data which indicated the existence of continuous pulmonary venous flow velocity >1.8 m/s, morphological features investigated with CTA, and intraoperative assessment (9). The preoperative obstruction included intrinsic stenosis that was identified when there was pulmonary ostial obstruction, pulmonary venous hypoplasia, or both; external obstruction that was identified when obstruction occurred within the anomalous connecting vein or at its connection to the systemic circulation; and restrictive atrial septal defect (<3 mm) defined when there was obstruction at the interatrial septum (6). Our data have 26 patients with and 22 without preoperative obstructive TAPVC. Early mortality was defined as death occurring during the hospital stay after the index operation.

### Blood Samples Processing

Each patient's peripheral blood was collected in ethylenediaminetetraacetic acid tube before operation and was stored at 4°C. Leukocytes were obtained after centrifugation and lysis of red blood cells, and then RNA was extracted by the TRIzol Reagent (Thermo Fisher, 10296028) following standard procedures as previously described (24). RNA concentration and integrity were evaluated by Agilent 2100 bioanalyzer (Agilent Technologies, G2939A). The sequencing library was prepared using PolyAdenylation Ligation Mediated-Seq (PALM-Seq) method, which depletes redundant rRNA and enriches informative RNA (25). Then, sequencing was performed on the DNBSEQ-T1 platform with 100 bp paired-end reads.

### Bioinformatic Processing of Sequencing Data

First of all, we carried out quality control on the raw sequencing data. Briefly, adapter, and low-quality reads were trimmed, then reads with > 10% ‘N' base or shorter than 17 bp were filtered by cutadapt (26). Reads aligned to rRNA were removed, mRNA and lncRNA from the GENCODE database (https://www.gencodegenes.org/). Bowtie was used to align all clean reads to transcripts references (27). The gene-level expression was estimated by RSEM (28). Principal component analysis (PCA) was calculated based on all genes through the R package “prcomp” function. Proportions of immune cell subtypes were calculated using CIBERSORT (29).

### Differential Expression Analysis

DEseq2 was used to analyze differential gene expression by setting age as a confounder (Supplementary Table 1), which adjusts for known batches using a linear model (30). Genes with log_2_ fold change (FC) ≥ 1 and the Bonferroni-Holm corrected *p* < 0.05 were defined as differentially expressed genes (DEG). We used clusterProfiler (V3.16.1) for gene enrichment of the up-regulated mRNA DEGs (31), based on the functional modules annotated by Gene Ontology (GO) (32), in which Bonferroni-Holm corrected *p*-value <0.05 were used as the threshold of significant enrichment. The heatmap was drawn by R package pheatmap. Single-cell expression data of the gene in leukocytes were obtained from the Human Cell Landscape (HCL) database (33).

### Quantitative Real-Time Reverse Transcription PCR (qRT-PCR)

Leukocyte RNA was reverse transcribed using an Evo M-MLV RT Premix for an RT-PCR kit (Accurate Biotechnology Co.Ltd, Hunan, China). Real-time PCR was carried out on a MyiQTM Single-Color Real-Time PCR detection system (Bio-Rad Laboratories, Hercules, CA, USA) with SYBR® Green Supermix (Bio-Rad Laboratories). Relative levels of expression in each assay were obtained by normalizing the Ct values of the tested genes against that of *GPADH*. The primer sequences used for mRNA or lncRNA expression are listed in Supplementary Table 2.

### Model Feature Selection

All patients with TAPVC are divided into training and validation sets. The training set of preoperative obstructive patients who cannot be identified by echocardiography or CTA The validation set of preoperative obstructive patients who were identified by echocardiography or CTA. There are 35 samples in the training set and 13 samples in the validation set, with a ratio of 7:3.

Next, we established a prediction model based on the training set in two steps. Step 1, data treating. To eliminate the effect of the order of magnitude and prevent the loss of feature information, we performed logarithmic transformation on all transcripts and standardized them to zero mean and standard deviation of one by scale function in R. Step 2, choose the most important features. We use generalized linear models (GLM) and random forests (RF) algorithms to select features based on the multiple iterations method. In each iteration, the GLM with seven-fold cross-validation (CV) was first used to select the top 100 features according to their importance, then the RF with seven-fold CV was used to select the top 50 features, and then use RF again to select the top five important features. After 20 iterations, we selected features that occurred more than 10 times as final features.

### Machine Learning Model Construction

To verify the usability of the features, we simultaneously build three algorithms for model construction, including GLM, RF, and support vector machine (SVM). All training workflows were performed in caret packages. Each model used seven-fold CV to select the hyperparameter, while ROC was an evaluation index to select the best hyperparameter. GLM used the glmnet package (V4.0–2) (34), while the alpha is set to 14 parameters 0–0.8 and lambda was set to 0–1. RF used the randomForest package (V4.6–14), (35) while the candidate hyperparameter of mtry was set to 1, 2, 3, 4. SVM used the kernlab package's svmLinear (V0.9–29) (36, 37), while the hyperparameter C was set to 11 parameters 0.0001–1.

AUROC calculation used the pROC package, the confusion matrix calculation used the caret package. After the hyperparameter selection, all training sets were used to refit the model. Finally, the best model was determined according to the area under the curve (AUC), Kappa, and F1 Scores in both training and validation cohorts. The flow chart is shown in Supplemental Figure 2.

### Statistical Analysis

Statistical analyses and data visualization were performed with R software (version 3.5.1). For continuous variables, median (range) was used to describe, and the Wilcoxon Mann–Whitney U test was used to compare differences between groups. Descriptive statistics for categorical variables were reported as frequency/percentage and were compared by Pearson χ2 or Fisher's exact test. Values of *p* < 0.05 were considered statistically significant.

## Results

### Clinical Information

In our data, 48 samples were included, of which 26 were obstructive and 22 were non-obstructive TAPVC patients. As shown in Table 1, the median surgical age was lower in the preoperative obstructive group (7.5 days) than those in the preoperative non-obstructive group (120.0 days) with statistical significance (*p* < 0.001). Similarly, there was also a difference in surgical weight between the two groups (median: 3.15 kg in the obstructive group vs. 5.25 kg in the non-obstructive group, *p* < 0.001). The distribution of the anatomy subtypes was not significantly different between the two groups (*p* = 0.13). Among the obstructive group, only 26.9% of obstructive patients could be identified by echocardiography or CTA scanning. The sutureless technique was more frequently used in the obstructive group (69.2 vs. 22.7% in the non-obstructive group, *p* < 0.001). The obstructive group had a longer duration of ventilation and stay in CCU post-operation. There was no significant difference in mortality between the two groups (*p* = 1.00).

**Table 1:** Clinical characteristics of preoperative obstructive patients and non-obstructive patients.

	**Preoperative obstructive patients (*n*= 26)**	**Preoperative** **non-obstructive patients (*n* = 22)**	***p***
Surgical age, d, median (Q1, Q3)	7.5 (3.0, 17.3)	120.0 (60.0, 1186.3)	<0.001
Male, *n* (%)	16 (61.5)	12 (54.5)	0.62
Surgical weight, kg, median (Q1, Q3)	3.15 (2.90, 3.40)	5.25 (4.45, 12.63)	<0.001
Prematurity, *n* (%)	2 (7.7)	2 (9.1)	1.00
Associated cardiac lesion, *n* (%)			
Patent ductus arteriosus	16 (61.5)	6 (27.3)	0.02
Atrial septal defect	26 (100)	22 (100)	1.00
Tricuspid insufficiency	8 (30.8)	17 (77.3)	0.00
Ventricular septal defect	1 (3.8)	0.00	1.00
Pulmonary artery stenosis	1 (3.8)	2 (9.1)	0.59
Coarctation of aorta	1 (3.8)	0.00	1.00
Mitrial insufficiency	3 (11.5)	2 (9.1)	1.00
Coronary artery pulmonary vein fistula	1 (3.8)	0.00	1.00
Pulmonary hypertenson	17 (51.5)	16 (48.5)	0.85
Anatomic type, n (%)			0.13
Supracardiac	15 (57.7)	10 (45.5)	
Cardiac	4 (15.4)	9 (40.9)	
Infracardiac	6 (23.0)	1 (4.5)	
Mixed	1 (3.8)	2 (9.1)	
PVO in CT/Echo examination/ oxygen saturation, n (%)	7 (26.9)	0.00	0.01
Preoperative poor status (intubation, heart failure, breath failure), n (%)	3 (11.5)	5 (22.7)	0.30
Emergency operation, n (%)	5 (19.2)	3 (13.6)	0.71
Use of sutureless repair, n (%)	18 (69.2)	5 (22.7)	<0.001
CPB time, min, median (Q1, Q3)	115 (84, 158)	102 (73, 122)	0.10
Aortic crossclamp time (min)	58 (47, 87)	54 (40, 67)	0.27
Postoperative conditions			
Duration of ventilation, h, median (Q1, Q3)	99 (52, 146)	52 (16, 124)	0.03
CCU stay, h, median (Q1, Q3)	4 (2, 5)	2 (2, 5)	0.28
Post-operative PVO, *n* (%)	2 (7.7)	1 (4.5)	0.18
Mortality, *n* (%)	2 (7.7)	1 (4.5)	1.00

### Transcript Profiling of Preoperative Obstructive and Non-obstructive TAPVC

**Figure 1:**
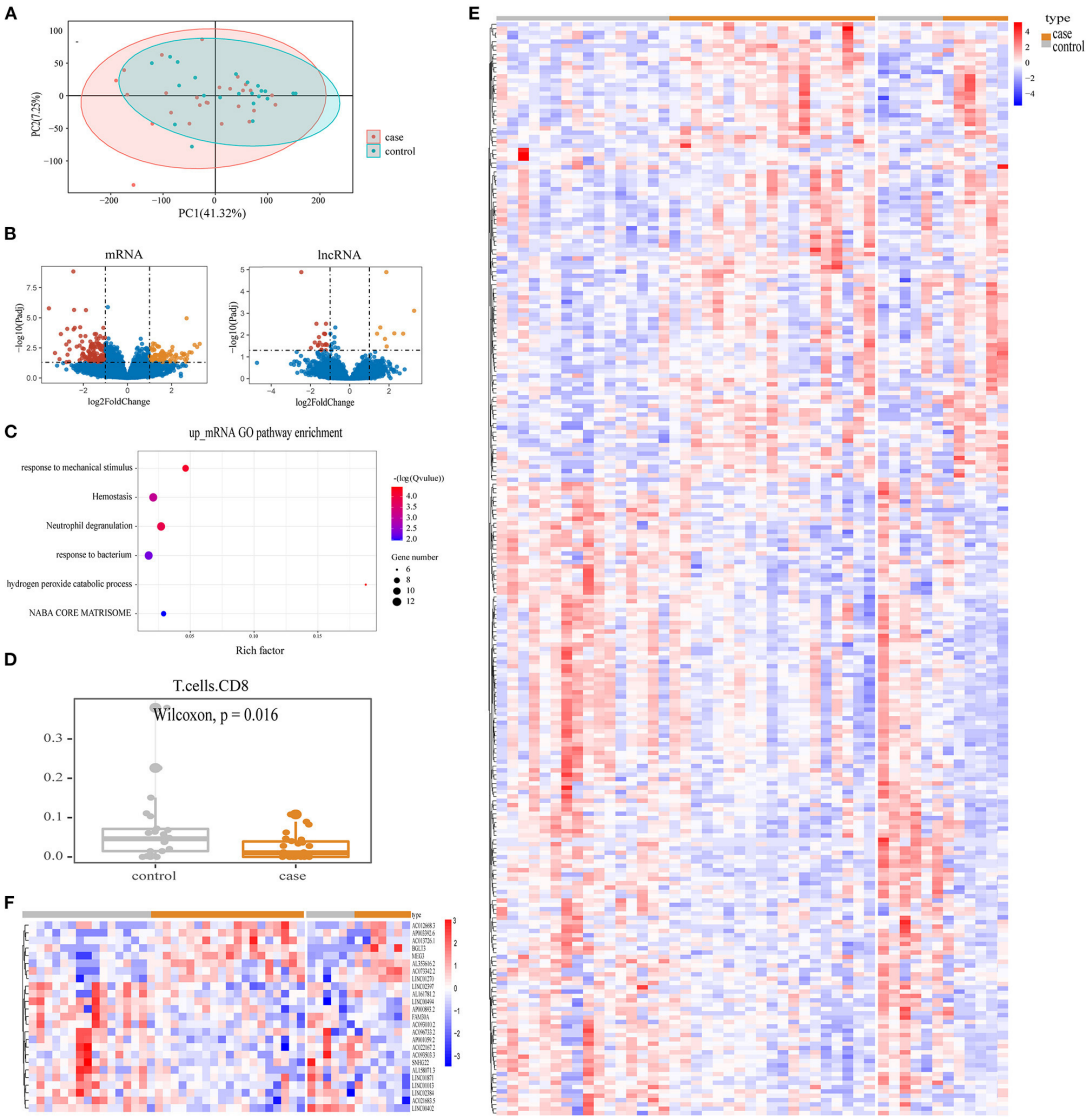
Enrichment results of obstructive TAPVC transcripts**. (A)** PCA map for all genes; **(B)** volcano map display of mRNA and lncRNA DEGs. Red indicates up-regulated genes; yellow indicates down-regulated genes; and blue indicates genes without significant changes; **(C)** GO terms for up-regulated mRNA; **(D)** percentage of CD8 T-cells; **(E)** heatmap of differentially expressed mRNA; **(F)** heatmap of differentially expressed lncRNA.

We successfully obtained the expression profiles of mRNA and lncRNA in 48 patients with TAPVC. To investigate whether the leukocyte transcriptome differed significantly between preoperative obstructive and non-obstructive TAPVC patients, we performed PCA analysis and found no significant differentiation (Figure 1A). To identify the obstructive-related characteristics, we performed differential expression analysis between obstructed and non-obstructed. As a result, we identified 256 mRNA and 27 lncRNA with altered abundance in blood, including 102 up-regulated and 154 down-regulated genes in the mRNA, 8 up-regulated and 19 down-regulated genes in the lncRNA (Figure 1B; Supplementary Tables 3, 4). Increased mRNA in obstructive patients enriched in 6 GO pathways (*p*-adjust <0.05), including hydrogen peroxide catabolic process, response to mechanical stimulus, neutrophil degranulation, hemostasis, response to the bacterium, and NABA CORE MATRISOME pathways (Figure 1C; Supplementary Table 5).

To investigate the effect of the immune cell subtype on preoperative obstruction, we performed a CIBERSORT analysis. The difference in the percentage of 22 immune cell types between obstructive and non-obstructive was subsequently compared and revealed a significant difference in the lower percentage of T-cells CD8 in preoperative obstructive TAPVC (*p* = 0.016), as shown in Figure 1D. DEG profiles were demonstrated based on heatmaps (Figures 1E,F).

### Construction of a Predictive Model for Preoperative Obstructive TAPVC

Differential expression analysis confirmed that leukocyte transcripts detected biologically relevant changes in patients with preoperative obstructive TAPVC. To assess whether leukocyte transcripts signatures could robustly classify patients with obstructive TAPVC, we build a preoperation prediction model. In the beginning, preoperative obstructive patients that could not be identified by echocardiography or CTA were classified as the training set (*n* = 19), and those that could be identified were grouped into the validation set (*n* = 7). Based on the surgery age ranking, the older preoperative non-obstructive patients were divided into the training set (*n* = 16) and the younger were divided into the validation set (*n* = 6). The ratio of the training set to the validation set is 7:3. Except for that surgical age (*p* = 0.017) and surgical weight (*p* = 0.043) were lower in the training set than those in the validation set with statistical significance, there were no significant differences in baseline characteristics between the two data sets (Supplementary Table 6). Similar to the finding from the total participants, the surgical age and surgical weight were significantly lower in obstructive patients than in non-obstructive patients in both data sets (*p* < 0.05).

**Figure 2:**
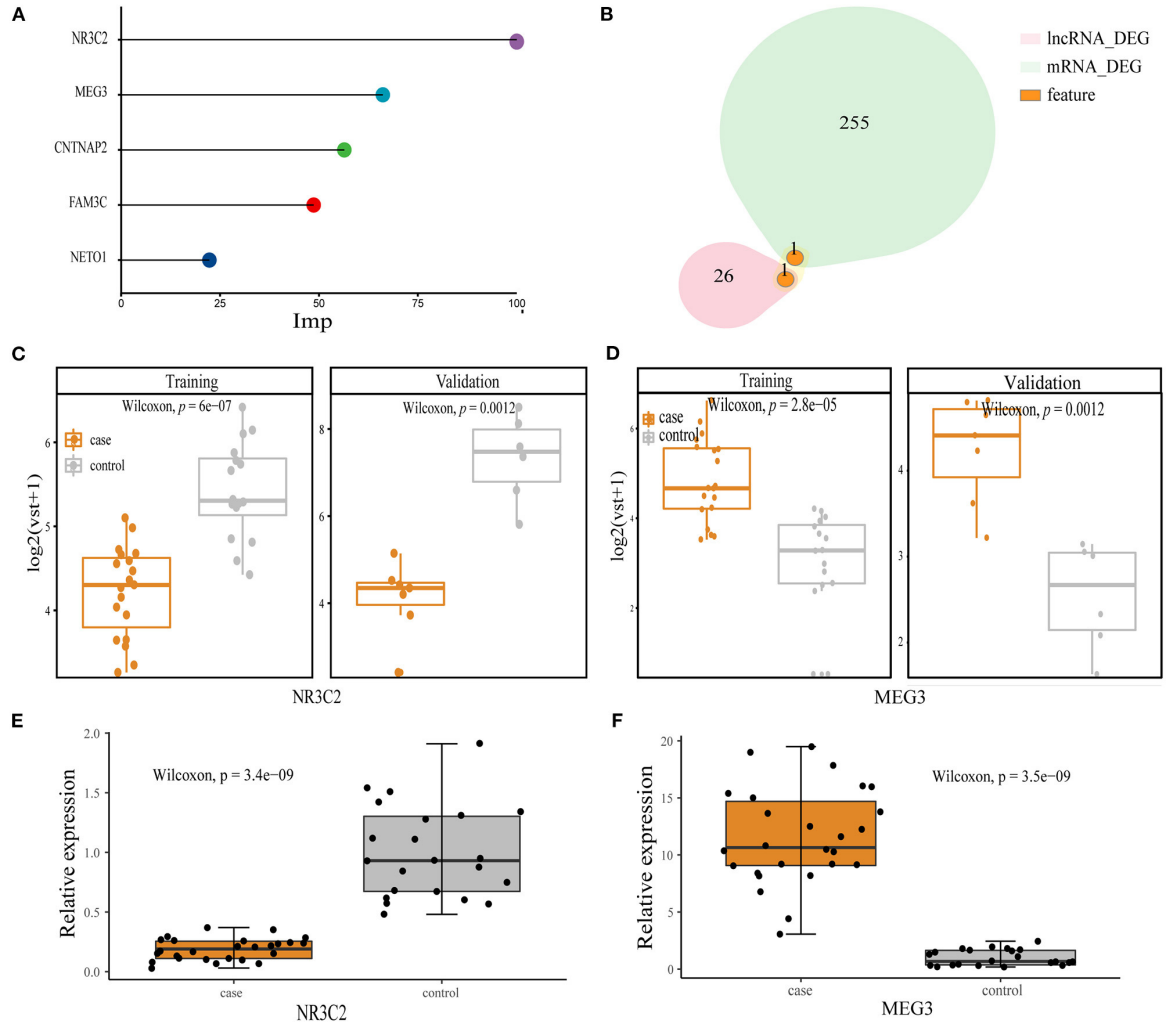
Model feature**. (A)** Top five features in order of importance; **(B)** overlap between *NR3C2, MEG3*, and mRNA, lncRNA DEGs; **(C,D)** the expression between the obstructive and non-obstructive TAPVC patients of *NR3C2* in the training set and the validation set, respectively, Wilcoxon-Mann-Whitney; **(C)** *NR3C2*; **(D)** *MEG3*; **(E)** qRT-PCR result for *NRC32*; **(F)** qRT-PCR result for *MEG3*.

We select features from all transcripts in the training set. The GLM and RF algorithms were used to calculate the importance of features based on seven-fold cv. After 20 iterations, we retain features of more than 10 repetitions, including mRNA *NR3C2, CNTNAP2, FAM3C, NET01*, and lncRNA *MEG3* (Figure 2A). *NR3C2* and *MEG3* were the two most important features and also the DEGs in the whole set (Figure 2B). We further compared the expression of these two genes in the training and validation sets, respectively. *NR3C2* was significantly down-expressed and *MEG3* was significantly up-expressed in patients with preoperative obstructive (Figures 2C,D). We verified the expression of *NR3C2* and *MEG3* by qRT-PCR, which showed the same trend as the transcriptome data and both of them are DEGs (Figures 2E,F).

**Figure 3:**
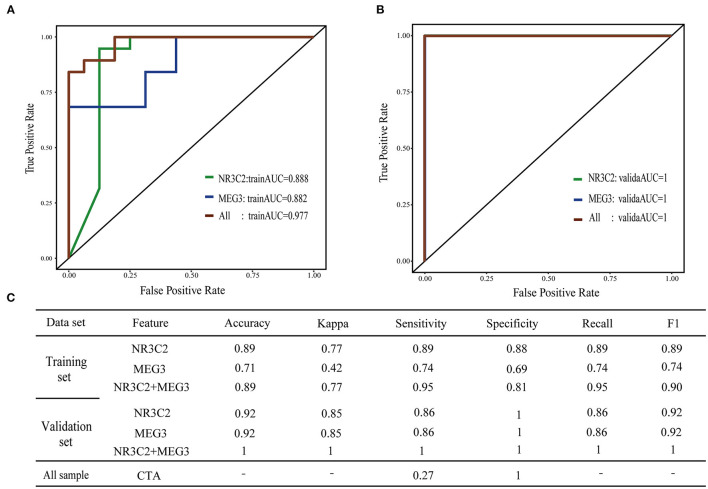
Machine learning model of preoperative obstructive TAPVC. **(A,B)** ROC curve of the training set and validation set; green indicates the *NR3C2* model; blue indicates the *MEG3* model; red indicates the combined model. **(A)** training set; **(B)** validation set; **(C)** evaluation index of the model.

To assess whether *NR3C2* and *MEG3* could robustly classify preoperative obstruction, the RF machine-learning was utilized to establish three models: the *NR3C2* model (model only included *NR3C2* feature), the *MEG3* model (model only included *MEG3* feature), and the combined model (model included both *NR3C2* and *MEG3*). The trainAUC and sensitivity were 0.89 and 0.89 in the *NR3C2* model, 0.89 and 0.74 in the *MEG3* model, and 0.98 and 0.95 in the combined model, respectively, in the training set. Consistently, the validaAUC and the sensitivity in the validation set were close to those in the training set (1.00 and 0.86 in the *NR3C2* model, 1.00 and 0.86 in the *MEG3* model, and 1.00 and 1.00 in the combined model, respectively) (Figures 3A,B). The other evaluation metrics of the models were shown in Figure 3C (Supplementary Table 7). To address the overfit bias, GLM and SVM machine-learning models were also established and achieved similar prediction performance (Supplementary Figure 3; Supplementary Table 8).

## Discussion

In the current study, we performed a transcripts comparison of peripheral leukocyte profiles between patients with obstructive and non-obstructive TAPVC. We found that the up-regulated mRNA in leukocytes were enriched in the hydrogen peroxide catabolic process, response to mechanical stimulus, neutrophil degranulation, hemostasis, response to the bacterium, and NABA CORE MATRISOME pathways, all of which are associated with the development of fibrosis. Leukocyte subtype analysis revealed a lower percentage of T-cells CD8 in preoperative obstructive TAPVC patients. The mRNA *NR3C2* and lncRNA *MEG3* were two features screened from all transcripts, which can correctly distinguish all patients with preoperative obstruction TAPVC in the validation set. Our project developed a novel imaging-independent approach, which is the first integration of peripheral blood leukocytes transcript to construct machine learning prediction models. It is expected to complement existing imaging techniques in clinical practice to guide the surgical protocol for TAPVC.

Venous vascular fibrosis leads to preoperative obstructive TAPVC (13, 14). The release of inflammatory mediators from fibroblast-activated venous triggers an influx of leukocytes to the site of injury, and the activated leukocytes further modulate pathological fibrosis (16). We found that up-regulated mRNAs of leukocytes in patients with preoperative obstructive TAPVC enriched in 6 GO pathways. Pathway 1, hydrogen peroxide catabolic process. ROS (including hydrogen peroxide) and oxidative stress appear to be important in renal fibrosis and tissue repair/remodeling after myocardial infarction (38). Pathway 2, response to mechanical stimulus. Fibroblasts are cells that respond to mechanical stretching stimuli. They maintain the structure and function of organ tissues by altering the expression of genes and proteins in their ECM in response to external physical, chemical, and biological factors (39). Pathway 3, neutrophil degranulation. Dysregulation of neutrophil degranulation capacity in patients with cystic fibrosis (CF) (40). Pathway 4, hemostasis. Hemostasis contributes to fibrosis of the lung, liver, kidney, and heart (41). Pathway 5, response to the bacterium. Bacterial respiratory infections are the main cause of morbidity and mortality in patients with cystic fibrosis (CF (42) Pathway 6, The NABA CORE MATRISOME pathway is a collection of genes encoding the core extracellular matrix, including ECM glycoproteins, collagens, and proteoglycans. The pathways that upregulate mRNA enrichment all seem to play a role in the development of fibrosis and the formation of ECM. In addition, we found the percentage of T-cell CD8 was lower in the preoperative obstructive TAPVC with significant differences. T-cell CD8was also found to negatively regulate renal fibrosis in previous experiments with knockout CD8 mice. These data suggest that gene dysregulation in the venous is reflected in the transcriptomic data of leukocytes.

The mRNA *NR3C2* and the lncRNA *MEG3* were the two most important features screened from all transcripts. *NR3C2*, the mineralocorticoid receptor, plays important role in the modulation of blood pressure (43). *MEG3*, Maternally expressed gene 3, is an imprinted gene (44). According to the HCL database, *NR3C2* is expressed in three subtypes of peripheral blood cells, including monocyte, T-cell CD8, and dendritic cell, while *MEG3* is expressed in the macrophage subtype (33). Dendritic cells are antigen-presenting cells that present captured antigens to T cells and regulate the number and activity of T-cell CD8 (45). T-cell CD8 then control the production of monocytes (46). Activated macrophages are derived from tissue-resident macrophages or peripheral blood monocytes and induce fibroblast activation by secreting TGF-β (47). And it has also been found that in the late stages of pulmonary fibrosis, activated macrophages and myofibroblasts are even thought to cross-stimulate each other, leading to a vicious circle that ensures the propagation of fibrosis (47). *MEG3* regulates TGF-β gene activity by binding to distal regulatory elements (48). We know that fibrosis is always marked by increased expression of TGF-β (49). We therefore hypothesized that *MEG3* in peripheral blood macrophages activates the expression of cytokines TGF-β, which promote fibrotic secretions by recruiting more fibroblasts, enhancing their differentiation to myofibroblasts, or promoting ECM proteins. We must acknowledge the limitations of peripheral blood leukocytes compared to diseased tissue cells in explaining disease mechanisms, and we need further studies to test this hypothesis and molecular mechanisms. However, peripheral blood leukocytes are an excellent material for noninvasive monitoring.

We constructed a machine-learning model to evaluate the predictive value of *NR3C2* and *MEG3*. The sensitivity achieved as high as one. However, the small-size dataset might lead to an overestimation of the model prediction performance. To reduce this effect, we used both RF and GLM algorithms to select features and constructed three machine learning prediction models RF, GLM, and SVM, all of which have good prediction results. However, a blinded clinical trial with a larger sample size and different races is essential before a diagnostic or screening test based on this work can be used in the clinic.

In our study, the patient information was collected from regional cardiac centers, which were internationally and nationally renowned, and the preoperative evaluation, imaging, and intraoperative exploration of patients are standardized, and the data collected are from medical records, which are more accurate and credible. But there are still limitations. First, since still no gold standard for the diagnosis, we can only define the disease using imaging and laboratory test results, as described in the methods section. It cannot be denied that there may be some preoperative obstructive TAPVC that fall outside these definitions, or that do not have a significant decrease in oxygen saturation at the time of presentation, which potentially leads to a misclassification bias of obstructive TAPVC in our study. Second, we observed variations in expression levels within groups. Although the patients with TAPVC recruited in the current study had similar baseline data, their complex etiologies and unclear and highly heterogeneous pathogenesis will unavoidably introduce variation within groups. The model lacks validation with external datasets, and we will further refine it in the subsequent study. Third, because of ethical issues, we were unable to directly obtain pulmonary vein tissue. Further validation using the pulmonary vein tissue is warranted based on ethical standards.

## Conclusion

Our pilot studies have shown that noninvasive blood tests were able to reflect the characteristics of preoperative obstruction in patients with TAPVC. mRNA *NR3C2* and lncRNA *MEG3* can accurately predict obstructive patients. Peripheral leukocyte RNA can be used as a biomarker source for noninvasive surveillance, but there are limitations in the interpretation of disease mechanisms. We believe that the application of blood transcripts will provide new research directions for disease research and help promote clinical development. Future research can integrate imaging, transcripts, and clinical features to find the most suitable clinically available indicators, which may truly change the preoperative monitoring of the disease.

## Data Availability Statement

The datasets presented in this study can be found in online repositories. The names of the repository/repositories and accession number(s) can be found below: http://db.cngb.org/cnsa, CNP0002105.

## Ethics Statement

The studies involving human participants were reviewed and approved by the Guangdong Provincial People's Hospital Human Subjects Committee (No. GDREC2018363H). Written informed consent to participate in this study was provided by the participants or their legal guardian/next of kin.

## Author Contributions

YO and W-JW conceptualized and designed the whole study. XiaohoL extracted white blood cells and conducted qRT-PCR verification. XiaohuL and HD are cardiac surgeons and cardiologists who were responsible for blood sample collection, disease diagnosis, and clinical information verification, and then the ZL isolated RNA and constructed PALM-Seq libraries. HQ reviewed the CTA data for patients and reconstructed the 3D image based on CTA data. ZW conducted preliminary analysis and drafted the first manuscript and JS, QZ, and TY verify the underlying data. ZW and XiaohoL critically revised the manuscript and YO and W-JW refined the manuscript. All authors contributed to the article and approved the submitted version.

## Funding

This work was supported by the National Key Research and Development Program [No. 2018YFC1002600], National Natural Science Foundation of China [Nos. 81903287 and 82170259], Natural Science Foundation of Guangdong Province [Nos. 2021A1515011445 and 2018A030313329], Guangdong Provincial Clinical Research Center for Cardiovascular disease [No. 2020B1111170011] and the Science, Technology, and Innovation Commission of Shenzhen Municipality under grant [No. JCYJ20180703093402288].

## Conflict of Interest

JS, ZL, QZ, JX, TY, and W-JW were employed by BGI-Shenzhen, Shenzhen, China. The remaining authors declare that the research was conducted in the absence of any commercial or financial relationships that could be construed as a potential conflict of interest.

## Publisher's Note

All claims expressed in this article are solely those of the authors and do not necessarily represent those of their affiliated organizations, or those of the publisher, the editors and the reviewers. Any product that may be evaluated in this article, or claim that may be made by its manufacturer, is not guaranteed or endorsed by the publisher.
