# Supplementary material for: Peripheral Blood Transcripts Predict Preoperative Obstructive Total Anomalous Pulmonary Venous Connection
Source: Front Cardiovasc Med. 2022 May 31;9:892000. doi: 10.3389/fcvm.2022.892000 (PMC9194086; doi:10.3389/fcvm.2022.892000)
Supplement: Supplementary file 1 [file Data_Sheet_1.docx]

Supplementary Material

**Supplementary Table 1**

**Supplementary Table 2**

**Supplementary Table 3**

**Supplementary Table 4**

**Supplementary Table 5**

**Supplementary Table 6**

**Supplementary Table 7**

**Supplementary Table 8**

**Supplementary Figure 1**

**Supplementary Figure 2**

**Supplementary Figure 3**

**Supplementary Table 1. Patient surgery age.**

| ID Surgery age |
| --- |
| T2004012126 10Y  T2004012127 28D  T2004012128 12D  T2004012129 4M  T2004012130 1M22d  T2004012131 3D  T2004012132 2M  T2004012133 7.5M  T2004012134 1H  T2004012135 3Y  T2004012137 2M  T2004012139 2.5M  T2004012140 9M  T2004012143 13D  T2004012144 2M  T2004012145 11D  T2004012146 4Y  T2004012147 4Y  T2004012149 6D  T2004012150 2M  T2004012151 3D  T2004012153 6D  T2004012154 7d  T2004012155 5M  T2004012156 5D  T2004012157 1D  T2004012158 2M  T2004012159 1M  T2004012160 11H  T2004012161 6D  T2004012162 2D  T2004012163 24D  T2004012164 1D  T2004012165 3M  T2004012166 48Y  T2004012167 2M23D  T2004012168 1D  T2004012169 8D  T2004012170 3M  T2004012174 15D  T2004012176 32Y  T2004012179 11D  T2004012180 3D  T2004012181 4.5M  T2004012182 3.5M  T2004012183 4.5M  T2004012185 15D  T2004012186 7M |

Y: year; M: month; D: day.

**Supplementary Table 2. PCR primers.**

| Gene-symbol | PCR primers (5′-3′) |
| --- | --- |
| NRC32 | F: GAGAAGGCTGGGGCTCATTT R: AGTGATGGCATGGACTGTGG |
| MEG3 | F: CTCCCCTTCTAGCGCTCACG  R: CTAGCCGCCGTCTATACTACCGGCT |

**Supplementary Table 3. Results of mRNA differential analysis.**

| GeneID | Length | control.Expression | case.Expression | log2FoldChange.case.control. | padj | Up.Down.Regulation | p.value |
| --- | --- | --- | --- | --- | --- | --- | --- |
| TSPEAR | 3963 | 2.220539 | 13.24483 | 2.576447 | 0.009713 | Up | 9.22E-05 |
| ORC3 | 2305.96 | 276.9101 | 855.5822 | 1.627489 | 0.009302 | Up | 8.66E-05 |
| MTRNR2L10 | 1530 | 304.9403 | 2140.998 | 2.811685 | 0.007269 | Up | 5.83E-05 |
| MTRNR2L1 | 1553 | 1538.793 | 5375.328 | 1.804554 | 0.045016 | Up | 0.001674 |
| HTR2A | 4689 | 2.4628 | 7.058112 | 1.518983 | 0.010153 | Up | 9.96E-05 |
| PIEZO2 | 5126.62 | 0.381117 | 2.205171 | 2.532585 | 0.011546 | Up | 0.000121 |
| JPH1 | 4378 | 0.62314 | 2.802608 | 2.169141 | 0.015616 | Up | 0.000214 |
| RELN | 1711.98 | 7.154532 | 22.1635 | 1.631256 | 0.007583 | Up | 6.16E-05 |
| HBG1 | 632.85 | 11131.26 | 99259.86 | 3.156594 | 0.002681 | Up | 1.11E-05 |
| KCNT2 | 5883 | 8.268781 | 23.95689 | 1.534694 | 0.011707 | Up | 0.000125 |
| HBE1 | 674.08 | 75.29896 | 346.5136 | 2.20221 | 0.011707 | Up | 0.000127 |
| HBG2 | 614 | 297404.3 | 1925589 | 2.694803 | 0.025345 | Up | 0.000513 |
| TK1 | 1628 | 22.63613 | 58.11905 | 1.360384 | 0.012405 | Up | 0.000146 |
| ELOVL6 | 4002.43 | 97.03684 | 233.1493 | 1.26465 | 0.012278 | Up | 0.00014 |
| SERPINI1 | 700.45 | 62.04049 | 149.0133 | 1.264159 | 0.011707 | Up | 0.000126 |
| OAT | 1997.91 | 574.2577 | 1455.49 | 1.341735 | 0.002155 | Up | 6.51E-06 |
| SLC16A1 | 2514.26 | 187.9278 | 416.2059 | 1.147119 | 0.018301 | Up | 0.000286 |
| IFIT1B | 1972 | 1320.261 | 3709.718 | 1.490486 | 0.019365 | Up | 0.000322 |
| THEM5 | 668.07 | 35.58447 | 114.8557 | 1.690503 | 0.049143 | Up | 0.002039 |
| HBZ | 755 | 140.7908 | 1360.454 | 3.272463 | 0.001474 | Up | 3.48E-06 |
| SLFN14 | 2889 | 263.004 | 613.912 | 1.222947 | 0.018679 | Up | 0.000301 |
| EDIL3 | 4475.14 | 193.9639 | 613.0647 | 1.660251 | 0.027944 | Up | 0.000627 |
| DPCD | 858 | 38.76331 | 82.63648 | 1.092087 | 0.022357 | Up | 0.000422 |
| HIGD1A | 1362.61 | 900.5877 | 1972.879 | 1.131364 | 0.002572 | Up | 9.89E-06 |
| FOSB | 3293.54 | 153.737 | 445.5016 | 1.534966 | 0.039643 | Up | 0.001235 |
| HNF4A | 6445 | 2.968305 | 9.445563 | 1.669997 | 0.015043 | Up | 0.0002 |
| DKK1 | 1790 | 0.340048 | 1.968884 | 2.533568 | 0.011546 | Up | 0.000122 |
| ZNF7 | 1927.68 | 177.2015 | 398.0934 | 1.167716 | 0.030603 | Up | 0.000739 |
| C10orf67 | 1486 | 2.365942 | 12.62292 | 2.415559 | 0.021824 | Up | 0.00041 |
| HSPA1B | 2521 | 534.6944 | 169.0703 | -1.66109 | 0.042242 | Down | 0.001409 |
| HSPH1 | 3373.38 | 759.5593 | 371.5158 | -1.03174 | 0.034091 | Down | 0.000944 |
| SERPINH1 | 989.54 | 42.13664 | 17.75295 | -1.24702 | 0.019445 | Down | 0.000325 |
| NPR3 | 661.73 | 33.008 | 127.3614 | 1.94804 | 0.041966 | Up | 0.001379 |
| AC000003.2 | 1447 | 15.08016 | 5.896901 | -1.35462 | 0.045906 | Down | 0.001754 |
| BPI | 1857.8 | 111.2258 | 277.476 | 1.318871 | 0.044997 | Up | 0.00164 |
| PRTN3 | 1026 | 12.46565 | 50.43309 | 2.016412 | 0.005065 | Up | 3.28E-05 |
| ELANE | 920 | 40.63615 | 122.9794 | 1.597581 | 0.041966 | Up | 0.001395 |
| MPO | 1986.88 | 141.2139 | 529.4454 | 1.9066 | 0.006675 | Up | 5.02E-05 |
| CHIT1 | 1959.62 | 7.094198 | 24.25448 | 1.77354 | 0.017662 | Up | 0.000269 |
| SLC28A3 | 4887 | 14.05645 | 41.91099 | 1.576096 | 0.0058 | Up | 4.11E-05 |
| RETN | 506.33 | 40.86227 | 129.4738 | 1.663819 | 0.010015 | Up | 9.63E-05 |
| GADD45A | 1130.43 | 336.1894 | 736.483 | 1.131378 | 0.005162 | Up | 3.37E-05 |
| HP | 1105.67 | 110.8295 | 333.0967 | 1.587599 | 0.046739 | Up | 0.001816 |
| CD177 | 2193.46 | 465.5849 | 2106.4 | 2.177663 | 0.044287 | Up | 0.001554 |
| ANXA3 | 1452.2 | 333.6273 | 908.5308 | 1.445298 | 0.012278 | Up | 0.00014 |
| ENTPD7 | 3550 | 75.22792 | 172.9403 | 1.200934 | 0.017213 | Up | 0.000258 |
| ATP9A | 7732.41 | 252.7623 | 509.3653 | 1.010919 | 0.01641 | Up | 0.000238 |
| SIGLEC5 | 2360 | 127.8249 | 280.8893 | 1.135833 | 0.027461 | Up | 0.000608 |
| ORM1 | 802 | 71.51239 | 213.8536 | 1.580358 | 0.015043 | Up | 0.0002 |
| B3GNT5 | 3804.3 | 591.1872 | 1404.222 | 1.248084 | 0.02261 | Up | 0.000431 |
| FCAR | 1268.27 | 583.1686 | 1237.637 | 1.085603 | 0.044362 | Up | 0.001598 |
| ETS2 | 2881.21 | 672.3409 | 1438.808 | 1.097609 | 0.044287 | Up | 0.001553 |
| FRMD4B | 3142.77 | 303.8866 | 677.7933 | 1.157312 | 0.001355 | Up | 2.50E-06 |
| ETV1 | 3282.86 | 0.316535 | 2.06606 | 2.706447 | 0.013061 | Up | 0.000162 |
| ZG16B | 716 | 2.380294 | 5.103506 | 1.100349 | 0.029415 | Up | 0.00068 |
| DES | 2248 | 0.91235 | 2.867786 | 1.652279 | 0.044149 | Up | 0.001518 |
| NDNF | 2885 | 2.121469 | 10.12416 | 2.254667 | 0.020773 | Up | 0.000376 |
| CHI3L1 | 1792 | 230.3636 | 620.7718 | 1.43015 | 0.002511 | Up | 8.54E-06 |
| PLSCR2 | 759 | 2.453987 | 6.097737 | 1.313146 | 0.04511 | Up | 0.001686 |
| BTNL8 | 1559.98 | 29.37071 | 60.14003 | 1.033947 | 0.003565 | Up | 1.67E-05 |
| TMTC1 | 4330.9 | 18.4889 | 55.50756 | 1.586025 | 0.048 | Up | 0.001957 |
| NOTCH3 | 5572.99 | 4.048777 | 12.22183 | 1.593902 | 0.025853 | Up | 0.000536 |
| LYVE1 | 2377.15 | 10.46047 | 31.29391 | 1.580934 | 0.00723 | Up | 5.75E-05 |
| NSUN7 | 3579.87 | 116.3345 | 243.8581 | 1.067763 | 0.047985 | Up | 0.001944 |
| MRVI1 | 5804.42 | 332.1427 | 772.9728 | 1.218614 | 0.004665 | Up | 2.91E-05 |
| DSC2 | 5204.73 | 1459.999 | 3442.006 | 1.237282 | 0.001474 | Up | 3.41E-06 |
| MYO10 | 4352.49 | 61.93948 | 146.7449 | 1.244379 | 0.020332 | Up | 0.000358 |
| RFX2 | 3180.8 | 151.7813 | 308.5341 | 1.023436 | 0.01641 | Up | 0.00024 |
| MGAM2 | 5503.73 | 231.7093 | 558.1568 | 1.268355 | 0.03116 | Up | 0.00077 |
| TLR5 | 3555.48 | 332.8176 | 709.6526 | 1.092381 | 0.044362 | Up | 0.001579 |
| PLB1 | 4483.35 | 87.32786 | 204.5201 | 1.227729 | 0.025752 | Up | 0.000526 |
| SH3PXD2B | 7032.78 | 37.61177 | 120.7323 | 1.682556 | 0.032431 | Up | 0.000838 |
| FLVCR2 | 2760.57 | 108.1554 | 231.558 | 1.098268 | 0.041725 | Up | 0.001356 |
| CXCL2 | 1218 | 7.349213 | 20.2135 | 1.459657 | 0.031358 | Up | 0.000783 |
| VSIG4 | 1682.63 | 13.19521 | 108.6148 | 3.041134 | 0.001798 | Up | 4.77E-06 |
| MGST1 | 903.34 | 76.16642 | 172.9232 | 1.182905 | 0.023982 | Up | 0.000475 |
| SLCO2B1 | 2395.28 | 1.116699 | 6.745786 | 2.594746 | 0.027188 | Up | 0.000591 |
| ME1 | 3371 | 17.20877 | 44.54377 | 1.37208 | 0.033392 | Up | 0.000874 |
| STAB1 | 4853.82 | 269.2631 | 719.3649 | 1.417707 | 0.012982 | Up | 0.000159 |
| OLFML2B | 2538.68 | 4.815243 | 19.39639 | 2.010108 | 0.014691 | Up | 0.000191 |
| MYO7A | 4075.16 | 9.583947 | 34.7687 | 1.859097 | 0.012373 | Up | 0.000144 |
| IGFBP2 | 846 | 2.860881 | 9.708626 | 1.762808 | 0.010153 | Up | 0.0001 |
| CTTNBP2 | 1458.53 | 1.395421 | 7.704649 | 2.465028 | 0.008864 | Up | 7.93E-05 |
| SASH1 | 6595.73 | 129.3878 | 305.508 | 1.239508 | 0.012154 | Up | 0.000136 |
| FN1 | 7414.63 | 20.64898 | 79.7511 | 1.949434 | 0.02965 | Up | 0.000698 |
| MRC1L1 | 5171 | 8.860688 | 38.30924 | 2.112202 | 0.027188 | Up | 0.00059 |
| IGF2BP1 | 4795.5 | 3.241811 | 13.30849 | 2.037475 | 0.002686 | Up | 1.13E-05 |
| C5orf67 | 369 | 0.933877 | 3.4243 | 1.874504 | 0.044149 | Up | 0.001516 |
| DGKI | 9873.33 | 3.000615 | 8.514841 | 1.504721 | 0.018719 | Up | 0.000304 |
| TIMP3 | 4603 | 6.693025 | 42.83819 | 2.678167 | 1.11E-05 | Up | 4.10E-09 |
| COL4A5 | 6427 | 1.790402 | 4.507398 | 1.332011 | 0.044149 | Up | 0.001519 |
| GABRE | 3152 | 2.432168 | 8.03772 | 1.724544 | 0.014691 | Up | 0.000191 |
| CHRNA2 | 2519.63 | 4.648811 | 11.39461 | 1.293418 | 0.024325 | Up | 0.000483 |
| SAMD14 | 2240.92 | 4.894385 | 11.14908 | 1.187725 | 0.020332 | Up | 0.00036 |
| PLCH1 | 6128 | 11.14972 | 24.70078 | 1.147549 | 0.020746 | Up | 0.000372 |
| NFIB | 7622 | 10.90405 | 28.37181 | 1.379593 | 0.002511 | Up | 8.85E-06 |
| CLEC1B | 933.62 | 71.96353 | 165.0502 | 1.197567 | 0.004226 | Up | 2.48E-05 |
| MRAP2 | 2153 | 1.21762 | 4.702213 | 1.949276 | 0.010712 | Up | 0.00011 |
| AC002996.1 | 784 | 2.893112 | 16.86884 | 2.543667 | 0.012154 | Up | 0.000135 |
| STK32B | 3241 | 3.092802 | 8.848437 | 1.516508 | 0.008449 | Up | 7.35E-05 |
| WFDC1 | 1509.29 | 0.390217 | 2.927673 | 2.907404 | 0.00773 | Up | 6.42E-05 |
| C5orf47 | 2522 | 2.11151 | 6.680898 | 1.661767 | 0.013923 | Up | 0.000175 |
| M1AP | 2457 | 1.171837 | 3.176837 | 1.438819 | 0.028242 | Up | 0.000637 |
| SMTN | 3008.14 | 7.474336 | 15.55957 | 1.057785 | 0.033392 | Up | 0.000875 |
| PGA4 | 840 | 0.951032 | 5.978951 | 2.652326 | 0.044362 | Up | 0.001592 |
| ECM1 | 2070 | 1.224301 | 3.374191 | 1.462583 | 0.04993 | Up | 0.002094 |
| ARHGEF28 | 4712.5 | 4.668752 | 1.395359 | -1.7424 | 0.038542 | Down | 0.001168 |
| FRMPD3 | 7155 | 7.124666 | 1.157946 | -2.62125 | 0.00428 | Down | 2.56E-05 |
| CXorf57 | 2880 | 6.122388 | 1.712527 | -1.83797 | 0.019559 | Down | 0.000335 |
| PTCH1 | 1026.72 | 56.70798 | 27.32149 | -1.05352 | 0.038845 | Down | 0.001197 |
| DLG3 | 4529 | 21.86638 | 10.06971 | -1.11869 | 0.018619 | Down | 0.000297 |
| JAKMIP1 | 2256 | 6.382885 | 1.536309 | -2.05474 | 0.012649 | Down | 0.000152 |
| XKRX | 2249 | 11.82216 | 4.029169 | -1.55294 | 0.002572 | Down | 9.74E-06 |
| DGKK | 7406 | 11.50408 | 2.556609 | -2.16984 | 0.033801 | Down | 0.000909 |
| XCL1 | 1367 | 9.176727 | 2.967543 | -1.62871 | 0.033976 | Down | 0.000928 |
| DERL3 | 1342.37 | 20.04157 | 8.86452 | -1.17688 | 0.020456 | Down | 0.000365 |
| GLDC | 574.12 | 15.09249 | 5.352287 | -1.4956 | 0.014329 | Down | 0.000183 |
| FCER2 | 1562.59 | 25.75148 | 12.38422 | -1.05615 | 0.015889 | Down | 0.000223 |
| LARGE2 | 1632.45 | 9.766735 | 3.992902 | -1.29044 | 0.044939 | Down | 0.00163 |
| CD19 | 1957 | 67.19857 | 29.26905 | -1.19905 | 0.002034 | Down | 5.90E-06 |
| CELSR1 | 11389 | 20.83274 | 10.41629 | -1.00001 | 0.012325 | Down | 0.000142 |
| FAM81A | 513 | 5.824188 | 2.574841 | -1.17757 | 0.019886 | Down | 0.000342 |
| NPR2 | 3686 | 6.019113 | 2.597116 | -1.21264 | 0.044997 | Down | 0.001647 |
| FCRL5 | 4613.43 | 142.0037 | 62.16938 | -1.19165 | 0.015079 | Down | 0.000205 |
| BHLHE41 | 3837 | 9.104531 | 3.048343 | -1.57856 | 0.030603 | Down | 0.000741 |
| PNOC | 1007 | 13.11674 | 6.449902 | -1.02406 | 0.013451 | Down | 0.000168 |
| ABCB4 | 2553.75 | 52.08455 | 23.77042 | -1.13169 | 0.003822 | Down | 2.01E-05 |
| FCRL2 | 1785.51 | 176.386 | 74.03675 | -1.25242 | 0.003162 | Down | 1.38E-05 |
| TPD52 | 2675.25 | 277.9757 | 129.576 | -1.10116 | 0.000477 | Down | 5.59E-07 |
| HS3ST1 | 3968.51 | 22.92398 | 9.786848 | -1.22794 | 0.00397 | Down | 2.18E-05 |
| VPREB3 | 575.84 | 52.55289 | 19.79362 | -1.40873 | 0.000478 | Down | 5.89E-07 |
| TCL1A | 1180.22 | 293.0173 | 120.5204 | -1.28171 | 0.001474 | Down | 3.23E-06 |
| SLAMF6 | 2738.11 | 391.8494 | 194.9012 | -1.00756 | 0.006675 | Down | 5.02E-05 |
| FCRL3 | 4725 | 303.8538 | 108.6002 | -1.48435 | 0.000226 | Down | 1.95E-07 |
| RARRES3 | 754.86 | 177.4212 | 77.129 | -1.20183 | 0.012649 | Down | 0.000152 |
| GZMK | 1509 | 174.2511 | 76.37759 | -1.18995 | 0.046945 | Down | 0.001835 |
| KLRK1 | 1480.4 | 581.2057 | 268.1782 | -1.11586 | 0.034731 | Down | 0.000985 |
| KLRB1 | 1448 | 257.5496 | 94.68677 | -1.44362 | 0.032431 | Down | 0.000837 |
| EOMES | 2080 | 58.3523 | 20.62716 | -1.50024 | 0.007081 | Down | 5.59E-05 |
| CXCR6 | 2048.25 | 28.56939 | 8.666527 | -1.72094 | 0.01641 | Down | 0.000237 |
| RNF165 | 7596 | 25.43231 | 10.36114 | -1.29548 | 0.020746 | Down | 0.000372 |
| SLC4A10 | 3398 | 71.76123 | 10.83357 | -2.7277 | 8.33E-05 | Down | 5.14E-08 |
| GPR68 | 2859 | 17.56052 | 7.905885 | -1.15134 | 0.045659 | Down | 0.001723 |
| PHLDB2 | 4354.49 | 53.68203 | 21.51762 | -1.31892 | 0.001937 | Down | 5.37E-06 |
| KIR3DL2 | 1546 | 12.07725 | 3.67838 | -1.71515 | 0.029306 | Down | 0.000673 |
| KIR2DL3 | 1590 | 13.88556 | 4.153068 | -1.74134 | 0.029306 | Down | 0.000674 |
| SH2D2A | 1599 | 41.83395 | 19.38587 | -1.10967 | 0.041496 | Down | 0.001338 |
| TIGIT | 3110.16 | 85.99813 | 42.59797 | -1.01352 | 0.017662 | Down | 0.00027 |
| KLRC2 | 1211 | 42.96977 | 12.25226 | -1.81027 | 0.02381 | Down | 0.000468 |
| SLC4A4 | 3771 | 30.19486 | 12.67238 | -1.25262 | 0.040726 | Down | 0.001298 |
| BNC2 | 12844 | 26.87596 | 9.774139 | -1.45927 | 0.044997 | Down | 0.001638 |
| MYBL1 | 5192 | 225.511 | 100.7129 | -1.16295 | 0.015067 | Down | 0.000202 |
| IL2RB | 4113 | 345.5395 | 154.8382 | -1.15809 | 0.041793 | Down | 0.001365 |
| TGFBR3 | 6186.63 | 267.8746 | 84.77491 | -1.65985 | 0.002511 | Down | 8.25E-06 |
| SH2D1B | 2523 | 129.0494 | 49.2583 | -1.38949 | 0.041057 | Down | 0.001317 |
| KLRF1 | 1215.81 | 220.2065 | 79.5952 | -1.4681 | 0.018432 | Down | 0.00029 |
| C1orf21 | 10265 | 108.4158 | 38.64736 | -1.48813 | 0.003223 | Down | 1.43E-05 |
| S1PR5 | 2191 | 89.28375 | 22.3333 | -1.9992 | 0.010712 | Down | 0.00011 |
| PRF1 | 1901.95 | 543.9684 | 163.4865 | -1.73435 | 0.006774 | Down | 5.18E-05 |
| SPON2 | 1869 | 165.6188 | 41.99853 | -1.97946 | 0.003822 | Down | 1.85E-05 |
| FCRL6 | 1246.4 | 44.39605 | 9.035953 | -2.29668 | 0.002511 | Down | 8.64E-06 |
| PRSS23 | 4015 | 38.74044 | 9.434624 | -2.0378 | 0.003471 | Down | 1.61E-05 |
| FGFBP2 | 1168 | 123.0802 | 32.44507 | -1.92353 | 0.015889 | Down | 0.000222 |
| GZMH | 909 | 141.2494 | 17.45612 | -3.01644 | 0.000226 | Down | 1.92E-07 |
| GNLY | 758.83 | 139.7281 | 46.11645 | -1.59927 | 0.020773 | Down | 0.00038 |
| KLRD1 | 4537.73 | 1082.107 | 516.6716 | -1.06652 | 0.005362 | Down | 3.57E-05 |
| DTHD1 | 2847.6 | 54.67791 | 17.75502 | -1.62273 | 0.011707 | Down | 0.000127 |
| PLEKHF1 | 2084 | 19.38735 | 6.275332 | -1.62735 | 0.010484 | Down | 0.000105 |
| CD160 | 3388 | 61.83273 | 14.89821 | -2.05323 | 0.00094 | Down | 1.48E-06 |
| CTSW | 1300 | 114.3209 | 47.1484 | -1.27781 | 0.020332 | Down | 0.000359 |
| TKTL1 | 2566 | 36.25762 | 16.55532 | -1.13099 | 0.01492 | Down | 0.000196 |
| EPS8L2 | 3148 | 8.324482 | 2.990397 | -1.47702 | 0.015889 | Down | 0.000224 |
| DUSP2 | 1688 | 218.9426 | 67.97173 | -1.68755 | 0.008887 | Down | 8.10E-05 |
| NKG7 | 673.24 | 424.2477 | 132.6976 | -1.67676 | 0.003465 | Down | 1.58E-05 |
| HOPX | 1093.76 | 81.88523 | 39.68423 | -1.04504 | 0.02733 | Down | 0.000603 |
| MYO6 | 5387 | 14.00621 | 4.387543 | -1.67458 | 0.012982 | Down | 0.00016 |
| LAG3 | 1775 | 12.36285 | 1.812094 | -2.77028 | 0.001653 | Down | 4.28E-06 |
| PPP2R2B | 3915.13 | 28.19521 | 9.649843 | -1.54687 | 0.008387 | Down | 7.24E-05 |
| ZNF683 | 1095.44 | 9.590635 | 0.814479 | -3.55768 | 1.64E-06 | Down | 3.03E-10 |
| ERICH3 | 4613.5 | 2.787239 | 0.751966 | -1.8901 | 0.02733 | Down | 0.000603 |
| MCOLN2 | 2871 | 64.11441 | 25.55636 | -1.32697 | 0.001369 | Down | 2.62E-06 |
| SLAMF7 | 2345 | 161.9828 | 75.25498 | -1.10598 | 0.018432 | Down | 0.000291 |
| IGF1 | 7359 | 10.96425 | 4.429649 | -1.30754 | 0.044205 | Down | 0.001529 |
| AC136428.1 | 349 | 24.21717 | 5.55581 | -2.12396 | 0.000324 | Down | 3.00E-07 |
| IGLL5 | 1070.67 | 323.8374 | 139.166 | -1.21846 | 0.046246 | Down | 0.001774 |
| TXNDC5 | 2964 | 533.9585 | 260.4857 | -1.03552 | 0.002511 | Down | 8.85E-06 |
| MZB1 | 825 | 24.45622 | 8.628388 | -1.50304 | 0.002511 | Down | 8.21E-06 |
| TNFRSF17 | 860.19 | 32.58358 | 12.51827 | -1.38011 | 0.040566 | Down | 0.001288 |
| NAP1L2 | 2550 | 5.243883 | 0.620739 | -3.07858 | 0.027461 | Down | 0.000613 |
| PLPPR4 | 4906.67 | 2.433371 | 0.358633 | -2.76238 | 0.048307 | Down | 0.001978 |
| SSPN | 2206.8 | 18.02737 | 3.371149 | -2.41888 | 2.23E-06 | Down | 5.51E-10 |
| TSHZ2 | 4968.96 | 38.68113 | 10.34719 | -1.90239 | 0.000334 | Down | 3.35E-07 |
| LTK | 2554.75 | 5.839581 | 1.744317 | -1.7432 | 0.023526 | Down | 0.000454 |
| NR3C2 | 3215.2 | 68.77372 | 12.56756 | -2.45215 | 1.43E-09 | Down | 8.79E-14 |
| KIF21A | 593 | 22.89628 | 4.222169 | -2.43906 | 9.31E-05 | Down | 6.31E-08 |
| DNAH8 | 13864 | 13.44087 | 2.567207 | -2.38835 | 6.86E-05 | Down | 3.78E-08 |
| PARM1 | 5011 | 17.43258 | 3.9509 | -2.14153 | 6.15E-05 | Down | 2.65E-08 |
| CNTNAP2 | 6434.7 | 27.39259 | 7.441149 | -1.88019 | 2.32E-06 | Down | 7.14E-10 |
| GPR15 | 1252 | 18.14221 | 7.530958 | -1.26845 | 0.039643 | Down | 0.001239 |
| SOX13 | 1129.02 | 18.52919 | 5.779935 | -1.68067 | 0.00094 | Down | 1.57E-06 |
| MS4A2 | 1936.22 | 79.83259 | 27.50559 | -1.53725 | 0.033801 | Down | 0.000915 |
| ITGB8 | 8751 | 58.41465 | 24.98869 | -1.22506 | 0.032069 | Down | 0.000809 |
| PCDH1 | 3827 | 10.30544 | 3.260034 | -1.66045 | 0.002572 | Down | 1.00E-05 |
| PTMS | 793.41 | 328.8915 | 153.314 | -1.10112 | 0.010426 | Down | 0.000104 |
| TSTA3 | 1328.28 | 481.8705 | 210.5985 | -1.19415 | 0.022717 | Down | 0.000437 |
| SMIM24 | 1004.12 | 80.12216 | 34.92294 | -1.19803 | 0.015889 | Down | 0.000224 |
| AC023632.1 | 1860 | 25.37126 | 9.868615 | -1.36228 | 0.031254 | Down | 0.000775 |
| CACNA1A | 4345.26 | 13.31892 | 4.600519 | -1.53361 | 0.038422 | Down | 0.001147 |
| FAM149A | 2997 | 6.154733 | 0.984401 | -2.64438 | 0.045016 | Down | 0.001679 |
| C1QTNF6 | 1346.7 | 28.59845 | 12.70523 | -1.17051 | 0.018826 | Down | 0.000306 |
| CADM1 | 4650.31 | 21.65617 | 7.35517 | -1.55795 | 0.004703 | Down | 2.96E-05 |
| HRASLS2 | 742 | 3.495725 | 0.531993 | -2.71611 | 0.011707 | Down | 0.000127 |
| SLC25A4 | 4440 | 9.67437 | 4.717382 | -1.03618 | 0.027188 | Down | 0.000593 |
| PDZD4 | 3418 | 6.778518 | 1.983814 | -1.77269 | 0.005935 | Down | 4.28E-05 |
| AGAP1 | 7212.68 | 15.69999 | 5.261536 | -1.57721 | 0.001474 | Down | 3.48E-06 |
| NAV3 | 2398 | 7.278423 | 2.622402 | -1.47274 | 0.018855 | Down | 0.000308 |
| GDF7 | 9749 | 5.583783 | 1.453347 | -1.94186 | 0.017662 | Down | 0.000269 |
| CD80 | 1662.33 | 7.104171 | 2.74415 | -1.37231 | 0.031878 | Down | 0.0008 |
| SLC38A11 | 2872.4 | 4.346739 | 1.588851 | -1.45195 | 0.033691 | Down | 0.000895 |
| TFAP2E | 2189 | 3.834573 | 1.660542 | -1.20741 | 0.038845 | Down | 0.001197 |
| WFS1 | 3255 | 9.42742 | 4.45837 | -1.08035 | 0.044362 | Down | 0.001576 |
| EDARADD | 1854 | 5.196808 | 2.051731 | -1.34078 | 0.043078 | Down | 0.001452 |
| LUM | 3008 | 7.087569 | 0.727966 | -3.28335 | 0.008491 | Down | 7.44E-05 |
| SAMD12 | 2172 | 26.00167 | 11.33814 | -1.19742 | 0.033475 | Down | 0.000886 |
| NRCAM | 3025 | 16.00768 | 3.945653 | -2.02043 | 0.009102 | Down | 8.36E-05 |
| C12orf42 | 1020.62 | 11.13887 | 5.379998 | -1.04993 | 0.047884 | Down | 0.001911 |
| RIC3 | 1435.89 | 32.98535 | 12.56841 | -1.39202 | 0.00094 | Down | 1.53E-06 |
| CYP2E1 | 1208 | 2.450645 | 0.583359 | -2.07071 | 0.02381 | Down | 0.000467 |
| WNT16 | 2188.5 | 3.122132 | 0.822079 | -1.92518 | 0.048189 | Down | 0.001968 |
| GLI1 | 3196 | 4.084582 | 1.665844 | -1.29394 | 0.049143 | Down | 0.002034 |
| CRIP3 | 465.94 | 3.961406 | 1.778436 | -1.1554 | 0.047884 | Down | 0.001903 |
| KLF8 | 3067.87 | 52.30646 | 23.61889 | -1.14705 | 0.002155 | Down | 6.39E-06 |
| PLPP5 | 1377.27 | 184.647 | 86.68412 | -1.09093 | 6.86E-05 | Down | 3.81E-08 |
| IFNLR1 | 4368.08 | 37.14777 | 15.75126 | -1.23781 | 0.000334 | Down | 3.50E-07 |
| ZCCHC18 | 2907 | 13.65633 | 6.388596 | -1.096 | 0.049607 | Down | 0.002074 |
| CD200 | 1182.66 | 61.81563 | 23.11543 | -1.41912 | 0.000226 | Down | 1.82E-07 |
| CCR6 | 3699.94 | 105.6668 | 40.00583 | -1.40124 | 0.043359 | Down | 0.001467 |
| SOX5 | 1947.61 | 15.23988 | 5.502718 | -1.46963 | 0.012349 | Down | 0.000143 |
| BEND4 | 6473.49 | 85.14501 | 42.05178 | -1.01775 | 0.000436 | Down | 4.83E-07 |
| CD72 | 1233.69 | 54.53692 | 24.17266 | -1.17386 | 0.004717 | Down | 3.02E-05 |
| BLK | 2334.57 | 106.8924 | 53.31871 | -1.00345 | 0.001408 | Down | 2.78E-06 |
| PAX5 | 7064.76 | 539.1547 | 218.0466 | -1.30606 | 0.000528 | Down | 6.84E-07 |
| MOXD1 | 2950 | 6.942126 | 2.216153 | -1.64732 | 0.001931 | Down | 5.24E-06 |
| KIF5C | 3502.23 | 14.16251 | 5.470599 | -1.37231 | 0.002641 | Down | 1.07E-05 |
| CDH2 | 563 | 7.371673 | 1.177986 | -2.64567 | 0.00094 | Down | 1.43E-06 |
| KLK2 | 528 | 17.67498 | 5.920505 | -1.57792 | 0.047504 | Down | 0.001869 |
| WNT7A | 4041 | 8.318695 | 3.95681 | -1.07202 | 0.044148 | Down | 0.001511 |
| MMP11 | 2291 | 5.057636 | 1.573778 | -1.68423 | 0.008887 | Down | 8.09E-05 |
| WNT3 | 3355 | 5.822484 | 1.29555 | -2.16807 | 0.002034 | Down | 5.77E-06 |
| DAZL | 3050 | 7.651512 | 2.740443 | -1.48134 | 0.047884 | Down | 0.001903 |
| PEG10 | 6529.66 | 58.15759 | 22.38307 | -1.37756 | 0.00939 | Down | 8.80E-05 |
| KCNH8 | 5137 | 29.71606 | 10.72454 | -1.47033 | 0.001474 | Down | 3.56E-06 |

**Supplementary Table 4. Results of lncRNA differential analysis.**

| GeneID | Length | control-Expression | case-Expression | log2Fold  Change  (case/control) | padj | Up/Down-Regulation | | p-value |
| --- | --- | --- | --- | --- | --- | --- | --- | --- |
| AC012668.3 | 788 | 1.635754 | 15.891811 | 3.280255831 | 0.00077 | Up | 2.73E-07 | |
| AC013726.1 | 2285 | 32.5402 | 85.511692 | 1.393898597 | 0.00867 | Up | 1.05E-05 | |
| AC021683.5 | 2722 | 40.977181 | 17.624111 | -1.217270244 | 0.04984 | Down | 0.000212 | |
| AC022167.2 | 1521.6 | 242.8056 | 43.988218 | -2.464612631 | 1.30E-05 | Down | 3.07E-09 | |
| AC073342.2 | 1800 | 17.937964 | 86.592758 | 2.27123023 | 0.00838 | Up | 7.90E-06 | |
| AC093010.2 | 3023 | 45.672634 | 19.034946 | -1.262679507 | 0.00877 | Down | 1.34E-05 | |
| AC093503.3 | 2352 | 35.438752 | 13.036139 | -1.442811134 | 0.04984 | Down | 0.000211 | |
| AC096733.2 | 1394 | 22.021128 | 7.5796522 | -1.538684831 | 0.02214 | Down | 5.15E-05 | |
| AL158071.3 | 506 | 3.8468877 | 0.9782092 | -1.975476727 | 0.03906 | Down | 0.000144 | |
| AL161781.2 | 739 | 8.6288618 | 3.2911964 | -1.39055817 | 0.02857 | Down | 8.44E-05 | |
| AL353616.2 | 1739 | 1.2576989 | 4.6411322 | 1.883690238 | 0.03344 | Up | 0.00011 | |
| AP000893.2 | 433 | 30.04299 | 12.586129 | -1.255193763 | 0.02857 | Down | 8.22E-05 | |
| AP001059.2 | 449 | 24.091208 | 8.9182084 | -1.433680895 | 0.04612 | Down | 0.000183 | |
| AP003392.6 | 325 | 1.6011666 | 5.5561184 | 1.794953905 | 0.01511 | Up | 3.03E-05 | |
| BGLT3 | 993 | 2.2312679 | 14.721037 | 2.721943706 | 0.00867 | Up | 1.23E-05 | |
| FAM30A | 2466.5 | 114.60775 | 49.545854 | -1.209868306 | 0.00304 | Down | 1.56E-06 | |
| LINC00402 | 3340 | 47.894777 | 16.141373 | -1.569105026 | 0.01245 | Down | 2.21E-05 | |
| LINC00494 | 2149.8 | 24.585056 | 11.041097 | -1.154898126 | 0.02857 | Down | 8.27E-05 | |
| LINC01013 | 1000.4 | 12.149022 | 3.7541172 | -1.694294628 | 0.00304 | Down | 1.79E-06 | |
| LINC01270 | 1893.9 | 26.762072 | 78.814612 | 1.558273316 | 0.0045 | Up | 3.72E-06 | |
| LINC01871 | 243 | 20.22572 | 6.6117261 | -1.613092166 | 0.02857 | Down | 8.76E-05 | |
| LINC02384 | 537.43 | 33.924638 | 14.613863 | -1.214995874 | 0.02598 | Down | 6.74E-05 | |
| LINC02397 | 10626 | 210.26945 | 87.747665 | -1.260806601 | 0.00904 | Down | 1.49E-05 | |
| MEG3 | 1998.5 | 7.9055635 | 28.811306 | 1.86569488 | 1.30E-05 | Up | 2.78E-09 | |
| MIR9-3HG | 1277.4 | 5.0603314 | 1.6486275 | -1.617966407 | 0.03079 | Down | 9.80E-05 | |
| SLC9A3-AS1 | 1011.1 | 39.221941 | 11.425164 | -1.779446089 | 0.02198 | Down | 4.66E-05 | |
| SNHG22 | 421 | 363.86341 | 146.75689 | -1.309968703 | 0.00867 | Down | 1.15E-05 | |

**Supplementary Table 5. Differentially expressed coding genes annotation.**

| Gene Symbol | Description | Protein Function (Protein Atlas) | |
| --- | --- | --- | --- |
| SH3PXD2B | SH3 and PX domains 2B | | Predicted intracellular proteins; Disease related genes |
| TMTC1 | transmembrane O-mannosyltransferase targeting cadherins 1 | |  |
| OLFML2B | olfactomedin like 2B | Predicted intracellular proteins; Predicted secreted proteins | |
| BPI | bactericidal permeability increasing protein | Transporters:Transporter channels and pores; Predicted intracellular proteins; Predicted secreted proteins | |
| PARM1 | prostate androgen-regulated mucin-like protein 1 |  | |
| C12orf42 | chromosome 12 open reading frame 42 | Predicted intracellular proteins | |
| ELANE | elastase, neutrophil expressed | Cancer-related genes:Candidate cancer biomarkers; FDA approved drug targets:Biotech drugs; Predicted secreted proteins; Peptidases:Serine-type peptidases; ENZYME proteins:Hydrolases; Enzymes; Disease related genes | |
| FOSB | FosB proto-oncogene, AP-1 transcription factor subunit | Predicted intracellular proteins; Transcription factors:Basic domains | |
| KLRK1 | killer cell lectin like receptor K1 | Cancer-related genes:Candidate cancer biomarkers; Transporters:Transporter channels and pores; CD markers | |
| FCAR | Fc fragment of IgA receptor | Predicted intracellular proteins; CD markers | |
| BNC2 | basonuclin 2 | Transcription factors:Zinc-coordinating DNA-binding domains; Predicted intracellular proteins | |
| PLAAT4 | phospholipase A and acyltransferase 4 | Predicted intracellular proteins | |
| RIC3 | RIC3 acetylcholine receptor chaperone | Predicted intracellular proteins; Transporters:Accessory Factors Involved in Transport | |
| GZMK | granzyme K | Predicted secreted proteins; Peptidases:Serine-type peptidases; Enzymes | |
| CCR6 | C-C motif chemokine receptor 6 | G-protein coupled receptors:Chemokines and chemotactic factors receptors; G-protein coupled receptors:GPCRs excl olfactory receptors; CD markers | |
| SLC38A11 | solute carrier family 38 member 11 | Transporters:Electrochemical Potential-driven transporters | |
| DAZL | deleted in azoospermia like | Predicted intracellular proteins | |
| DGKI | diacylglycerol kinase iota | ENZYME proteins:Transferases; Predicted intracellular proteins; Enzymes | |
| PLEKHF1 | pleckstrin homology and FYVE domain containing 1 | Predicted intracellular proteins | |
| MMP11 | matrix metallopeptidase 11 | Cancer-related genes:Candidate cancer biomarkers; Predicted secreted proteins; FDA approved drug targets:Small molecule drugs; Enzymes; Peptidases:Metallopeptidases | |
| TSPEAR | thrombospondin type laminin G domain and EAR repeats | Predicted intracellular proteins; Predicted secreted proteins; Disease related genes | |
| WNT16 | Wnt family member 16 | Predicted intracellular proteins; Predicted secreted proteins | |
| SLC28A3 | solute carrier family 28 member 3 | Transporters:Electrochemical Potential-driven transporters | |
| DGKK | diacylglycerol kinase kappa | ENZYME proteins:Transferases; Predicted intracellular proteins; Enzymes | |
| SH2D2A | SH2 domain containing 2A | Predicted intracellular proteins | |
| PLSCR2 | phospholipid scramblase 2 | Predicted intracellular proteins; Transporters | |
| M1AP | meiosis 1 associated protein | Predicted intracellular proteins | |
| FCRL2 | Fc receptor like 2 | CD markers | |
| MS4A2 | membrane spanning 4-domains A2 | Transporters:Transporter channels and pores; Predicted intracellular proteins; FDA approved drug targets:Biotech drugs | |
| MGAM2 | maltase-glucoamylase 2 (putative) | ENZYME proteins:Hydrolases; Enzymes | |
| FCER2 | Fc fragment of IgE receptor II | Cancer-related genes:Candidate cancer biomarkers; Predicted intracellular proteins; Predicted secreted proteins; CD markers | |
| KIR3DL2 | killer cell immunoglobulin like receptor, three Ig domains and long cytoplasmic tail 2 | CD markers | |
| BTNL8 | butyrophilin like 8 |  | |
| PPP2R2B | protein phosphatase 2 regulatory subunit Bbeta | Predicted intracellular proteins; Disease related genes | |
| PRF1 | perforin 1 | Predicted intracellular proteins; Disease related genes; Cancer-related genes; Transporters:Transporter channels and pores; Potential drug targets | |
| FRMPD3 | FERM and PDZ domain containing 3 | Predicted intracellular proteins | |
| LUM | lumican | Cancer-related genes:Mutational cancer driver genes; Predicted secreted proteins | |
| GADD45A | growth arrest and DNA damage inducible alpha | Cancer-related genes:Candidate cancer biomarkers; Predicted intracellular proteins | |
| PGA4 | pepsinogen A4 | Predicted intracellular proteins; Predicted secreted proteins; ENZYME proteins:Hydrolases; Enzymes; Peptidases:Aspartic-type peptidases | |
| IL2RB | interleukin 2 receptor subunit beta | Cancer-related genes:Candidate cancer biomarkers; CD markers; Transporters; FDA approved drug targets:Biotech drugs; Predicted intracellular proteins | |
| PIEZO2 | piezo type mechanosensitive ion channel component 2 | Transporters:Transporter channels and pores; Predicted intracellular proteins; Potential drug targets; Disease related genes | |
| DES | desmin | Cancer-related genes:Candidate cancer biomarkers; Predicted intracellular proteins; Disease related genes | |
| XKRX | XK related X-linked | Transporters:Electrochemical Potential-driven transporters | |
| NKG7 | natural killer cell granule protein 7 | Predicted intracellular proteins | |
| None | None | None | |
| A1BG | alpha-1-B glycoprotein | Predicted intracellular proteins; Predicted secreted proteins | |
| DUSP2 | dual specificity phosphatase 2 | Predicted intracellular proteins; ENZYME proteins:Hydrolases; Enzymes | |
| IGKV2D-29 | immunoglobulin kappa variable 2D-29 |  | |
| SIGLEC5 | sialic acid binding Ig like lectin 5 |  | |
| CTTNBP2 | cortactin binding protein 2 | Predicted intracellular proteins | |
| IGLL5 | immunoglobulin lambda like polypeptide 5 | Predicted intracellular proteins; Predicted secreted proteins | |
| MTRNR2L10 | MT-RNR2 like 10 | Predicted intracellular proteins; Predicted secreted proteins | |
| DPCD | deleted in primary ciliary dyskinesia homolog (mouse) | Predicted intracellular proteins | |
| TGFBR3 | transforming growth factor beta receptor 3 | Cancer-related genes:Candidate cancer biomarkers; Predicted secreted proteins | |
| ECM1 | extracellular matrix protein 1 | Predicted secreted proteins; Disease related genes | |
| KLRD1 | killer cell lectin like receptor D1 | Predicted intracellular proteins; CD markers | |
| ORM1 | orosomucoid 1 | Cancer-related genes:Candidate cancer biomarkers; Candidate cardiovascular disease genes; Predicted secreted proteins | |
| CELSR1 | cadherin EGF LAG seven-pass G-type receptor 1 | Transporters; G-protein coupled receptors:Family 2 (B) receptors; Predicted intracellular proteins; G-protein coupled receptors:GPCRs excl olfactory receptors; Disease related genes; Potential drug targets | |
| VSIG4 | V-set and immunoglobulin domain containing 4 |  | |
| SLFN14 | schlafen family member 14 | Predicted intracellular proteins; Disease related genes | |
| VPREB3 | V-set pre-B cell surrogate light chain 3 | Predicted intracellular proteins | |
| GDF7 | growth differentiation factor 7 | Predicted secreted proteins | |
| PLPP5 | phospholipid phosphatase 5 | ENZYME proteins:Hydrolases; Enzymes | |
| GPR15 | G protein-coupled receptor 15 | G-protein coupled receptors:GPCRs excl olfactory receptors | |
| IGF2BP1 | insulin like growth factor 2 mRNA binding protein 1 | Predicted intracellular proteins | |
| SSPN | sarcospan |  | |
| HSPH1 | heat shock protein family H (Hsp110) member 1 | Cancer-related genes:Candidate cancer biomarkers; Predicted intracellular proteins | |
| C5orf67 | chromosome 5 putative open reading frame 67 |  | |
| TFAP2E | transcription factor AP-2 epsilon | Predicted intracellular proteins; Transcription factors:Basic domains | |
| DSC2 | desmocollin 2 | Disease related genes | |
| GZMH | granzyme H | Predicted secreted proteins; Peptidases:Serine-type peptidases; Enzymes | |
| CD177 | CD177 molecule | Predicted intracellular proteins | |
| HNF4A | hepatocyte nuclear factor 4 alpha | Transcription factors:Zinc-coordinating DNA-binding domains; Nuclear receptors; Disease related genes; Predicted intracellular proteins | |
| ETS2 | ETS proto-oncogene 2, transcription factor | Predicted intracellular proteins; Transcription factors:Helix-turn-helix domains; RAS pathway related proteins | |
| MYBL1 | MYB proto-oncogene like 1 | Predicted intracellular proteins; Transcription factors:Helix-turn-helix domains | |
| GFUS | GDP-L-fucose synthase | Predicted intracellular proteins; Enzymes; ENZYME proteins:Oxidoreductases | |
| SLAMF6 | SLAM family member 6 | CD markers | |
| CRIP3 | cysteine rich protein 3 | Predicted intracellular proteins | |
| PDZD4 | PDZ domain containing 4 | Cancer-related genes:Candidate cancer biomarkers; Predicted intracellular proteins | |
| DLG3 | discs large MAGUK scaffold protein 3 | Predicted intracellular proteins; Disease related genes | |
| FAM149A | family with sequence similarity 149 member A | Predicted intracellular proteins | |
| WFS1 | wolframin ER transmembrane glycoprotein | Potential drug targets; Disease related genes; Transporters:Accessory Factors Involved in Transport | |
| JPH1 | junctophilin 1 |  | |
| None | None | None | |
| FN1 | fibronectin 1 | Cancer-related genes:Candidate cancer biomarkers; Candidate cardiovascular disease genes; FDA approved drug targets:Biotech drugs; Predicted intracellular proteins; Predicted secreted proteins; Disease related genes; Cancer-related genes:Mutational cancer driver genes | |
| AGAP1 | ArfGAP with GTPase domain, ankyrin repeat and PH domain 1 | Predicted intracellular proteins | |
| CTSW | cathepsin W | Peptidases:Cysteine-type peptidases; Enzymes; Predicted secreted proteins | |
| FCRL6 | Fc receptor like 6 | Predicted intracellular proteins | |
| NAV3 | neuron navigator 3 | Cancer-related genes:Mutational cancer driver genes; Disease related genes; Predicted intracellular proteins | |
| SOX13 | SRY-box transcription factor 13 | Predicted intracellular proteins; Transcription factors:Other all-alpha-helical DNA-binding domains | |
| NR3C2 | nuclear receptor subfamily 3 group C member 2 | Transcription factors:Zinc-coordinating DNA-binding domains; Predicted intracellular proteins; FDA approved drug targets:Small molecule drugs; Disease related genes; Nuclear receptors | |
| EPS8L2 | EPS8 like 2 | Predicted intracellular proteins; Disease related genes | |
| B3GNT5 | UDP-GlcNAc:betaGal beta-1,3-N-acetylglucosaminyltransferase 5 | ENZYME proteins:Transferases; Predicted intracellular proteins; Enzymes | |
| TCL1A | TCL1 family AKT coactivator A | Predicted intracellular proteins; Disease related genes; Cancer-related genes | |
| ABCB4 | ATP binding cassette subfamily B member 4 | Predicted intracellular proteins; Transporters:Primary Active Transporters; ENZYME proteins:Hydrolases; Enzymes; Disease related genes; Potential drug targets | |
| WFDC1 | WAP four-disulfide core domain 1 | Predicted secreted proteins | |
| MRAP2 | melanocortin 2 receptor accessory protein 2 | Disease related genes | |
| SLC25A4 | solute carrier family 25 member 4 | Transporters:Electrochemical Potential-driven transporters; FDA approved drug targets:Small molecule drugs; Disease related genes | |
| SOX5 | SRY-box transcription factor 5 | Predicted intracellular proteins; Transcription factors:Other all-alpha-helical DNA-binding domains; Disease related genes | |
| RELN | reelin | Predicted intracellular proteins; Predicted secreted proteins; Disease related genes | |
| NAP1L2 | nucleosome assembly protein 1 like 2 | Predicted intracellular proteins | |
| OAT | ornithine aminotransferase | ENZYME proteins:Transferases; Predicted intracellular proteins; Enzymes; Disease related genes; Potential drug targets | |
| MTRNR2L1 | MT-RNR2 like 1 | Predicted intracellular proteins; Predicted secreted proteins | |
| PLB1 | phospholipase B1 | Predicted intracellular proteins; ENZYME proteins:Hydrolases; Enzymes | |
| KLF8 | Kruppel like factor 8 | Transcription factors:Zinc-coordinating DNA-binding domains; Predicted intracellular proteins | |
| SMIM24 | small integral membrane protein 24 | Predicted intracellular proteins | |
| PNOC | prepronociceptin | Predicted intracellular proteins; Predicted secreted proteins | |
| S1PR5 | sphingosine-1-phosphate receptor 5 | Predicted intracellular proteins; FDA approved drug targets:Small molecule drugs; G-protein coupled receptors:Lysolipids receptors; G-protein coupled receptors:GPCRs excl olfactory receptors | |
| KIF21A | kinesin family member 21A | Predicted intracellular proteins; Disease related genes | |
| FLVCR2 | FLVCR heme transporter 2 | Predicted intracellular proteins; Transporters:Electrochemical Potential-driven transporters; Potential drug targets; Disease related genes | |
| MZB1 | marginal zone B and B1 cell specific protein | Predicted secreted proteins | |
| SLC4A10 | solute carrier family 4 member 10 | Transporters:Electrochemical Potential-driven transporters | |
| HBG1 | hemoglobin subunit gamma 1 | Predicted intracellular proteins | |
| HBE1 | hemoglobin subunit epsilon 1 | Predicted intracellular proteins | |
| FCRL3 | Fc receptor like 3 | Predicted intracellular proteins; Disease related genes; CD markers | |
| JAKMIP1 | janus kinase and microtubule interacting protein 1 | Predicted intracellular proteins | |
| SPON2 | spondin 2 | Predicted intracellular proteins; Predicted secreted proteins | |
| SAMD12 | sterile alpha motif domain containing 12 | Predicted intracellular proteins | |
| CHIT1 | chitinase 1 | Candidate cardiovascular disease genes; Predicted secreted proteins; ENZYME proteins:Hydrolases; Enzymes | |
| PEG10 | paternally expressed 10 | Predicted intracellular proteins | |
| SLC4A4 | solute carrier family 4 member 4 | Predicted intracellular proteins; Transporters:Electrochemical Potential-driven transporters; Potential drug targets; Disease related genes | |
| LTK | leukocyte receptor tyrosine kinase | ENZYME proteins:Transferases; Kinases:Tyr protein kinases; Predicted intracellular proteins; Enzymes; Disease related genes; Potential drug targets | |
| TIMP3 | TIMP metallopeptidase inhibitor 3 | Cancer-related genes:Candidate cancer biomarkers; Predicted secreted proteins; Disease related genes | |
| NDNF | neuron derived neurotrophic factor | Predicted secreted proteins | |
| DERL3 | derlin 3 | Predicted intracellular proteins | |
| HTR2A | 5-hydroxytryptamine receptor 2A | G-protein coupled receptors:Serotonin receptors; FDA approved drug targets:Small molecule drugs; G-protein coupled receptors:GPCRs excl olfactory receptors | |
| STK32B | serine/threonine kinase 32B | ENZYME proteins:Transferases; Predicted intracellular proteins; Kinases; Enzymes | |
| MYO10 | myosin X | Predicted intracellular proteins | |
| IGKV2D-28 | immunoglobulin kappa variable 2D-28 |  | |
| PLPPR4 | phospholipid phosphatase related 4 | ENZYME proteins:Hydrolases; Enzymes | |
| ERICH3 | glutamate rich 3 | Predicted intracellular proteins | |
| IFNLR1 | interferon lambda receptor 1 | Predicted intracellular proteins | |
| SLC16A1 | solute carrier family 16 member 1 | Transporters:Electrochemical Potential-driven transporters; Potential drug targets; Disease related genes | |
| BLK | BLK proto-oncogene, Src family tyrosine kinase | Cancer-related genes:Candidate cancer biomarkers; ENZYME proteins:Transferases; Kinases:Tyr protein kinases; Predicted intracellular proteins; Enzymes; Disease related genes; Potential drug targets | |
| RADX | RPA1 related single stranded DNA binding protein, X-linked | Predicted intracellular proteins | |
| MYO7A | myosin VIIA | Predicted intracellular proteins; Disease related genes | |
| CHRNA2 | cholinergic receptor nicotinic alpha 2 subunit | Predicted intracellular proteins; FDA approved drug targets:Small molecule drugs; Disease related genes | |
| NPR3 | natriuretic peptide receptor 3 | Predicted intracellular proteins | |
| CD72 | CD72 molecule | Predicted intracellular proteins; CD markers | |
| GNLY | granulysin | Transporters:Transporter channels and pores; Predicted intracellular proteins; Predicted secreted proteins | |
| FGFBP2 | fibroblast growth factor binding protein 2 | Predicted secreted proteins | |
| PAX5 | paired box 5 | Cancer-related genes:Candidate cancer biomarkers; Predicted intracellular proteins; Disease related genes; Transcription factors:Helix-turn-helix domains | |
| FRMD4B | FERM domain containing 4B | Predicted intracellular proteins | |
| MPO | myeloperoxidase | Cancer-related genes:Candidate cancer biomarkers; Candidate cardiovascular disease genes; Cancer-related genes:Mutated cancer genes; Predicted intracellular proteins; ENZYME proteins:Oxidoreductases; Disease related genes; Enzymes; Potential drug targets | |
| None | None | None | |
| HIGD1A | HIG1 hypoxia inducible domain family member 1A |  | |
| KLRB1 | killer cell lectin like receptor B1 | CD markers | |
| NFIB | nuclear factor I B | Predicted intracellular proteins; Cancer-related genes; Transcription factors:beta-Hairpin exposed by an alpha/beta-scaffold | |
| FCRL5 | Fc receptor like 5 | Disease related genes; Predicted secreted proteins; CD markers | |
| PTMS | parathymosin | Predicted intracellular proteins | |
| GLDC | glycine decarboxylase | Predicted intracellular proteins; Enzymes; ENZYME proteins:Oxidoreductases; Disease related genes; Potential drug targets | |
| GLI1 | GLI family zinc finger 1 | Transcription factors:Zinc-coordinating DNA-binding domains; Predicted intracellular proteins | |
| CHI3L1 | chitinase 3 like 1 | Cancer-related genes:Candidate cancer biomarkers; Predicted intracellular proteins; Predicted secreted proteins; Disease related genes | |
| IGHV3-72 | immunoglobulin heavy variable 3-72 |  | |
| KCNH8 | potassium voltage-gated channel subfamily H member 8 | Voltage-gated ion channels:Voltage-Gated Potassium Channels | |
| PHLDB2 | pleckstrin homology like domain family B member 2 | Predicted intracellular proteins | |
| KLK2 | kallikrein related peptidase 2 | Cancer-related genes:Candidate cancer biomarkers; Predicted intracellular proteins; Predicted secreted proteins; Peptidases:Serine-type peptidases; ENZYME proteins:Hydrolases; Enzymes | |
| PRSS23 | serine protease 23 | Predicted intracellular proteins; Predicted secreted proteins; Peptidases:Serine-type peptidases; Enzymes | |
| RETN | resistin | Candidate cardiovascular disease genes; Predicted secreted proteins | |
| HBZ | hemoglobin subunit zeta | Predicted intracellular proteins | |
| NSUN7 | NOP2/Sun RNA methyltransferase family member 7 | Predicted intracellular proteins | |
| SAMD14 | sterile alpha motif domain containing 14 | Predicted intracellular proteins | |
| ENTPD7 | ectonucleoside triphosphate diphosphohydrolase 7 |  | |
| CADM1 | cell adhesion molecule 1 | Predicted intracellular proteins | |
| SERPINI1 | serpin family I member 1 | Predicted intracellular proteins; Predicted secreted proteins; Disease related genes | |
| SLCO2B1 | solute carrier organic anion transporter family member 2B1 | Predicted intracellular proteins; FDA approved drug targets:Small molecule drugs; Transporters:Electrochemical Potential-driven transporters | |
| DTHD1 | death domain containing 1 | Predicted intracellular proteins | |
| DKK1 | dickkopf WNT signaling pathway inhibitor 1 | Predicted secreted proteins | |
| ME1 | malic enzyme 1 | Predicted intracellular proteins; Enzymes; ENZYME proteins:Oxidoreductases | |
| TKTL1 | transketolase like 1 | ENZYME proteins:Transferases; Predicted intracellular proteins; Enzymes | |
| PLCH1 | phospholipase C eta 1 | Predicted intracellular proteins; ENZYME proteins:Hydrolases; Enzymes | |
| SERPINH1 | serpin family H member 1 | Predicted intracellular proteins; Disease related genes | |
| RNF165 | ring finger protein 165 | ENZYME proteins:Transferases; Predicted intracellular proteins; Enzymes | |
| C5orf47 | chromosome 5 open reading frame 47 | Predicted intracellular proteins | |
| TSHZ2 | teashirt zinc finger homeobox 2 | Predicted intracellular proteins; Transcription factors:Helix-turn-helix domains | |
| MCOLN2 | mucolipin TRP cation channel 2 | Transporters:Transporter channels and pores; Voltage-gated ion channels:Transient Receptor Potential Channels | |
| LARGE2 | LARGE xylosyl- and glucuronyltransferase 2 | Predicted intracellular proteins | |
| TIGIT | T cell immunoreceptor with Ig and ITIM domains |  | |
| GPR68 | G protein-coupled receptor 68 | Potential drug targets; G-protein coupled receptors:GPCRs excl olfactory receptors; Disease related genes | |
| MRC1 | mannose receptor C-type 1 | FDA approved drug targets:Small molecule drugs; CD markers | |
| ZNF683 | zinc finger protein 683 | Transcription factors:Zinc-coordinating DNA-binding domains; Predicted intracellular proteins | |
| ZG16B | zymogen granule protein 16B | Predicted intracellular proteins; Predicted secreted proteins | |
| IGF1 | insulin like growth factor 1 | Predicted secreted proteins; Disease related genes; RAS pathway related proteins | |
| CXCL2 | C-X-C motif chemokine ligand 2 | Cancer-related genes:Candidate cancer biomarkers; Predicted secreted proteins | |
| ETV1 | ETS variant transcription factor 1 | Predicted intracellular proteins; Disease related genes; Cancer-related genes; Transcription factors:Helix-turn-helix domains | |
| CD160 | CD160 molecule | Predicted intracellular proteins; CD markers | |
| COL4A5 | collagen type IV alpha 5 chain | Cancer-related genes:Candidate cancer biomarkers; Predicted intracellular proteins; Predicted secreted proteins; Disease related genes | |
| KIF5C | kinesin family member 5C | Predicted intracellular proteins; Disease related genes | |
| HS3ST1 | heparan sulfate-glucosamine 3-sulfotransferase 1 | ENZYME proteins:Transferases; Predicted intracellular proteins; Enzymes | |
| ARHGEF28 | Rho guanine nucleotide exchange factor 28 | Predicted intracellular proteins | |
| LYVE1 | lymphatic vessel endothelial hyaluronan receptor 1 |  | |
| MYO6 | myosin VI | Predicted intracellular proteins; Disease related genes | |
| KIR2DL3 | killer cell immunoglobulin like receptor, two Ig domains and long cytoplasmic tail 3 | CD markers | |
| ITGB8 | integrin subunit beta 8 | Cancer-related genes:Candidate cancer biomarkers | |
| PLAAT2 | phospholipase A and acyltransferase 2 |  | |
| CD19 | CD19 molecule | Disease related genes; CD markers; FDA approved drug targets:Biotech drugs | |
| ZCCHC18 | zinc finger CCHC-type containing 18 | Predicted intracellular proteins | |
| PRTN3 | proteinase 3 | Candidate cardiovascular disease genes; Predicted intracellular proteins; Predicted secreted proteins; Peptidases:Serine-type peptidases; ENZYME proteins:Hydrolases; Enzymes; Disease related genes; Potential drug targets | |
| CDH2 | cadherin 2 | Predicted intracellular proteins; CD markers | |
| CD200 | CD200 molecule | Predicted intracellular proteins; CD markers | |
| DNAH8 | dynein axonemal heavy chain 8 | Predicted intracellular proteins | |
| EDIL3 | EGF like repeats and discoidin domains 3 | Predicted secreted proteins | |
| TXNDC5 | thioredoxin domain containing 5 | Predicted intracellular proteins | |
| PCDH1 | protocadherin 1 | Predicted intracellular proteins | |
| IRAG1 | inositol 1,4,5-triphosphate receptor associated 1 | Predicted intracellular proteins | |
| STAB1 | stabilin 1 |  | |
| WNT7A | Wnt family member 7A | Predicted intracellular proteins; Predicted secreted proteins; Disease related genes | |
| NRCAM | neuronal cell adhesion molecule | Predicted intracellular proteins | |
| TNFRSF17 | TNF receptor superfamily member 17 | CD markers; Predicted intracellular proteins; Disease related genes; Cancer-related genes | |
| CD80 | CD80 molecule | Predicted intracellular proteins; CD markers; FDA approved drug targets:Biotech drugs | |
| PTCH1 | patched 1 | Cancer-related genes:Candidate cancer biomarkers; Predicted intracellular proteins; Transporters:Electrochemical Potential-driven transporters; Disease related genes; Potential drug targets | |
| ANXA3 | annexin A3 | Predicted intracellular proteins; FDA approved drug targets:Small molecule drugs | |
| WNT3 | Wnt family member 3 | Predicted secreted proteins; Disease related genes | |
| FAM81A | family with sequence similarity 81 member A | Predicted intracellular proteins | |
| ORC3 | origin recognition complex subunit 3 | Predicted intracellular proteins | |
| TLR5 | toll like receptor 5 | Predicted intracellular proteins; Disease related genes | |
| MGST1 | microsomal glutathione S-transferase 1 | ENZYME proteins:Transferases; Predicted intracellular proteins; Enzymes | |
| KCNT2 | potassium sodium-activated channel subfamily T member 2 | Transporters:Transporter channels and pores; Voltage-gated ion channels:Calcium-Activated Potassium Channels; Predicted intracellular proteins | |
| TK1 | thymidine kinase 1 | Cancer-related genes:Candidate cancer biomarkers; Predicted intracellular proteins; ENZYME proteins:Transferases; Enzymes | |
| HOPX | HOP homeobox | Predicted intracellular proteins; Transcription factors:Helix-turn-helix domains | |
| C1orf21 | chromosome 1 open reading frame 21 | Predicted intracellular proteins | |
| IFIT1B | interferon induced protein with tetratricopeptide repeats 1B | Predicted intracellular proteins | |
| XCL1 | X-C motif chemokine ligand 1 | Predicted secreted proteins | |
| ELOVL6 | ELOVL fatty acid elongase 6 | ENZYME proteins:Transferases; Enzymes | |
| IGFBP2 | insulin like growth factor binding protein 2 | Cancer-related genes:Candidate cancer biomarkers; Predicted intracellular proteins; Predicted secreted proteins | |
| HBG2 | hemoglobin subunit gamma 2 | Predicted intracellular proteins; Disease related genes | |
| LAG3 | lymphocyte activating 3 | Predicted secreted proteins; CD markers | |
| SH2D1B | SH2 domain containing 1B | Predicted intracellular proteins | |
| ZNF7 | zinc finger protein 7 | Transcription factors:Zinc-coordinating DNA-binding domains; Predicted intracellular proteins | |
| C1QTNF6 | C1q and TNF related 6 | Predicted intracellular proteins; Predicted secreted proteins | |
| BHLHE41 | basic helix-loop-helix family member e41 | Predicted intracellular proteins; Transcription factors:Basic domains | |
| CYP2E1 | cytochrome P450 family 2 subfamily E member 1 | Cancer-related genes:Candidate cancer biomarkers; Predicted intracellular proteins; Enzymes; ENZYME proteins:Oxidoreductases | |
| CLEC1B | C-type lectin domain family 1 member B | Predicted intracellular proteins | |
| EOMES | eomesodermin | Predicted intracellular proteins; Transcription factors:Immunoglobulin fold; Disease related genes | |
| EDARADD | EDAR associated death domain | Predicted intracellular proteins; Disease related genes | |
| THEM5 | thioesterase superfamily member 5 | Predicted intracellular proteins; ENZYME proteins:Hydrolases; Enzymes | |
| C10orf67 | chromosome 10 open reading frame 67 | Predicted intracellular proteins | |
| BEND4 | BEN domain containing 4 | Predicted intracellular proteins | |
| None | None | None | |
| A2M | alpha-2-macroglobulin | Cancer-related genes:Candidate cancer biomarkers; Candidate cardiovascular disease genes; Predicted secreted proteins | |
| RFX2 | regulatory factor X2 | Predicted intracellular proteins; Transcription factors:Helix-turn-helix domains | |
| GABRE | gamma-aminobutyric acid type A receptor subunit epsilon | FDA approved drug targets:Small molecule drugs | |
| ATP9A | ATPase phospholipid transporting 9A (putative) | Transporters:Primary Active Transporters; ENZYME proteins:Hydrolases; Enzymes | |
| None | None | None | |
| MOXD1 | monooxygenase DBH like 1 | Predicted intracellular proteins | |
| CNTNAP2 | contactin associated protein 2 | Predicted intracellular proteins; Disease related genes | |
| TPD52 | tumor protein D52 | Cancer-related genes:Candidate cancer biomarkers; Predicted intracellular proteins | |
| CXCR6 | C-X-C motif chemokine receptor 6 | G-protein coupled receptors:Chemokines and chemotactic factors receptors; G-protein coupled receptors:GPCRs excl olfactory receptors; CD markers | |
| SASH1 | SAM and SH3 domain containing 1 | Predicted intracellular proteins | |
| HSPA1B | heat shock protein family A (Hsp70) member 1B | Predicted intracellular proteins | |
| SLAMF7 | SLAM family member 7 | Predicted intracellular proteins; CD markers; FDA approved drug targets:Biotech drugs | |
| NOTCH3 | notch receptor 3 | Cancer-related genes:Candidate cancer biomarkers; Predicted intracellular proteins; Disease related genes | |
| KLRF1 | killer cell lectin like receptor F1 |  | |
| KLRC2 | killer cell lectin like receptor C2 | Predicted intracellular proteins; CD markers | |
| HP | haptoglobin | Cancer-related genes:Candidate cancer biomarkers; Predicted intracellular proteins; Predicted secreted proteins; Peptidases:Serine-type peptidases; Enzymes; Disease related genes; Potential drug targets | |
| SMTN | smoothelin | Predicted intracellular proteins | |
| NPR2 | natriuretic peptide receptor 2 | ENZYME proteins:Lyases; Transporters:Accessory Factors Involved in Transport; Predicted intracellular proteins; FDA approved drug targets:Small molecule drugs; Enzymes; Disease related genes; Kinases:RGC receptor guanylate cyclase kinases | |
| CACNA1A | calcium voltage-gated channel subunit alpha1 A | Voltage-gated ion channels:Voltage-Gated Calcium Channels; Predicted intracellular proteins; FDA approved drug targets:Small molecule drugs; Disease related genes; Transporters:Transporter channels and pores | |

**Supplementary Table 6. Clinical features between the training and validation set.**

|  | Training | | | validation | | | Trainging versus Validation |
| --- | --- | --- | --- | --- | --- | --- | --- |
|  | obstructive patients (n=19) | non-obstructive patients (n=16) | *p*-value | obstructive patients (n=7) | non-obstructive patients (n=6) | *p*-value | *p*-value |
| PVO in CTA, n (%) | 0 | 0 |  | 7 (100) | 0 | 0.001 | <0.001 |
| Surgical age, d, median (Q1, Q3) | 7.00 (3.0, 15.0) | 79.0 (60.0, 146.3) | 0.001 | 13 (3.0, 120.0) | 2555.0 (1368.8, 13140.0) | 0.003 | 0.017 |
| Male, n (%) | 13 (68.4) | 8 (50.0) | 0.268 | 3 (42.9) | 4 (66.7) | 0.592 | 0.701 |
| Surgical weight, kg, median (Q1, Q3) | 3.1 (2.90, 3.40) | 4.85 (4.08, 5.60) | 0.001 | 3.25 (2.90, 4.50) | 18.0 (12.88, 50.0) | 0.003 | 0.043 |
| Prematurity, n (%) | 1 (5.3) | 2 (12.5) | 1.000 | 1 (14.3) | 0 | 1.000 | 1.000 |
| Associated cardiac lesion |  |  |  |  |  |  |  |
| Patent ductus arteriosus | 12 (63.2) | 6 (37.5) | 0.130 | 4 (57.1) | 0 | 0.070 | 0.202 |
| Atrial septal defect | 19 (100) | 16 (100) | 1.000 | 7 (100) | 6 (100) | 1.000 | 1.000 |
| Tricuspid Insufficiency | 5 (26.3) | 12 (75.0) | 0.004 | 3 (42.9) | 5 (83.3) | 0.266 | 0.424 |
| Ventricular septal defect | 1 (5.3) | 0 | 1.000 | 0 | 0 | 1.000 | 1.000 |
| Pulmonary artery stenosis | 1 (5.3) | 0 | 1.000 | 0 | 2 (33.3) | 0.192 | 0.174 |
| Coarctation of aorta | 0 | 0 | 1.000 | 1 (14.3) | 0 | 1.000 | 0.271 |
| Mitrial Insufficiency | 2 (10.5) | 0 | 0.489 | 1 (14.3) | 2 (33.3) | 0.559 | 0.115 |
| Coronary artery pulmonary vein fistula | 1 (5.3) | 0 | 1.000 | 0 | 0 | 1.000 | 1.000 |
| Pulmonary Hypertenson | 10 (52.6) | 11 (68.8) | 0.332 | 7 (100.0) | 5 (83.3) | 0.462 | 0.073 |
| Anatomic type |  |  | 0.128 |  |  | 0.316 | 0.386 |
| Supracardiac | 9 (47.4) | 6 (37.5) |  | 6 (85.7) | 4 (66.7) |  |  |
| Cardiac | 4 (21.1) | 7 (43.8) |  | 0 | 2 (33.3) |  |  |
| Infracardiac | 5 (26.4) | 1 (6.3) |  | 1 (14.3) | 0 |  |  |
| Mixed | 1 (5.3) | 2 (12.5) |  | 0 | 0 |  |  |

**Supplementary Table 7. RF predictive model evaluation metrics.**

|  | Training | Validation |
| --- | --- | --- |
| pnrn | 13 | 6 |
| pyrn | 3 | 0 |
| pnry | 1 | 0 |
| pyry | 18 | 7 |
| Accuracy | 0.885714286 | 1 |
| Kappa | 0.76744186 | 1 |
| AccuracyLower | 0.732621956 | 0.752947362 |
| AccuracyUpper | 0.967968831 | 1 |
| AccuracyNull | 0.542857143 | 0.538461538 |
| AccuracyPValue | 1.59E-05 | 0.000319898 |
| McnemarPValue | 0.617075077 | NaN |
| Sensitivity | 0.947368421 | 1 |
| Specificity | 0.8125 | 1 |
| Pos Pred Value | 0.857142857 | 1 |
| Neg Pred Value | 0.928571429 | 1 |
| Precision | 0.857142857 | 1 |
| Recall | 0.947368421 | 1 |
| F1 | 0.9 | 1 |
| Prevalence | 0.542857143 | 0.538461538 |
| Detection Rate | 0.514285714 | 0.538461538 |
| Detection Prevalence | 0.6 | 0.538461538 |
| Balanced Accuracy | 0.879934211 | 1 |
| trainROC | 0.976973684 |  |
| KFmeanAUC | 1 |  |
| predAUC |  | 1 |

The first four rowspnrns are confusion matrix: predict no and real no; pyrn: predict yes but real no; pnry: predict no but real yes; pyry: predict yes and real yes.

**Supplementary table 8. Other predictive models evaluation metrics.**

|  | SVM_model | | GLM_model | |
| --- | --- | --- | --- | --- |
|  | Training | Validation | Training | Validation |
| pnrn | 16 | 6 | 11 | 6 |
| pyrn | 0 | 0 | 5 | 0 |
| pnry | 4 | 3 | 0 | 0 |
| pyry | 15 | 4 | 19 | 7 |
| Accuracy | 0.8857143 | 0.769231 | 0.85714286 | 1 |
| Kappa | 0.7741935 | 0.551724 | 0.70489039 | 1 |
| AccuracyLower | 0.732622 | 0.461868 | 0.69742865 | 0.75294736 |
| AccuracyUpper | 0.9679688 | 0.949619 | 0.95193922 | 1 |
| AccuracyNull | 0.5428571 | 0.538462 | 0.54285714 | 0.53846154 |
| AccuracyPValue | 1.59E-05 | 0.079832 | 8.7043E-05 | 0.0003199 |
| McnemarPValue | 0.1336144 | 0.248213 | 0.07363827 | NaN |
| Sensitivity | 0.7894737 | 0.571429 | 1 | 1 |
| Specificity | 1 | 1 | 0.6875 | 1 |
| Pos Pred Value | 1 | 1 | 0.79166667 | 1 |
| Neg Pred Value | 0.8 | 0.666667 | 1 | 1 |
| Precision | 1 | 1 | 0.79166667 | 1 |
| Recall | 0.7894737 | 0.571429 | 1 | 1 |
| F1 | 0.8823529 | 0.727273 | 0.88372093 | 1 |
| Prevalence | 0.5428571 | 0.538462 | 0.54285714 | 0.53846154 |
| Detection Rate | 0.4285714 | 0.307692 | 0.54285714 | 0.53846154 |
| Detection Prevalence | 0.4285714 | 0.307692 | 0.68571429 | 0.53846154 |
| Balanced Accuracy | 0.8947368 | 0.785714 | 0.84375 | 1 |
| trainROC | 0.9736842 |  | 0.97697368 |  |
| KFmeanAUC | 0.5595238 |  | 0.96428571 |  |
| predAUC |  | 1 |  | 1 |

The first four rowspnrns are confusion matrix: pnrn: predict no and real no; pyrn: predict yes but real no; pnry: predict no but real yes; pyry: predict yes and real yes.


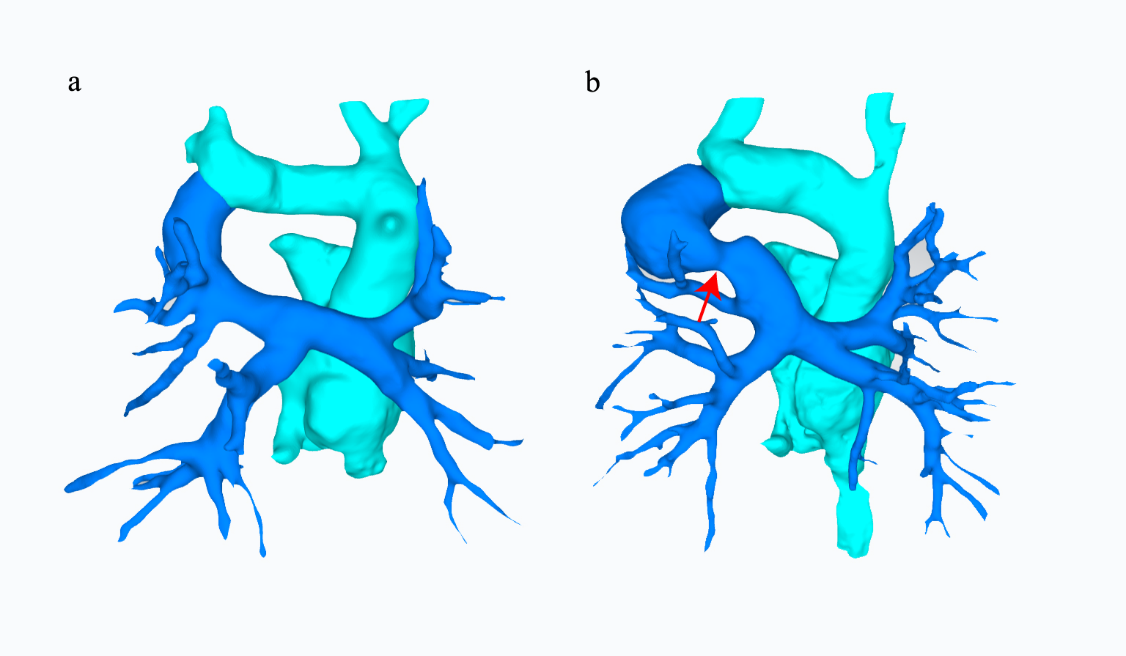


**Supplementary Figure 1.** 3D reconstruction based on CTA images of preoperative non-obstructive and obstructive TAPVC. a preoperative non-obstructive TAPVC. b preoperative obstructive TAPVC. The arrow marks the obstructive position.


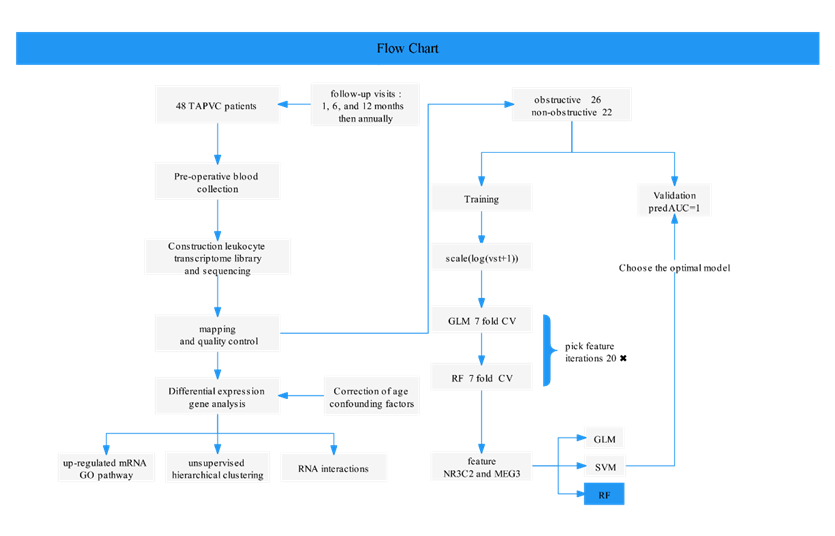


**Supplementary Figure 2.** Flow chart.


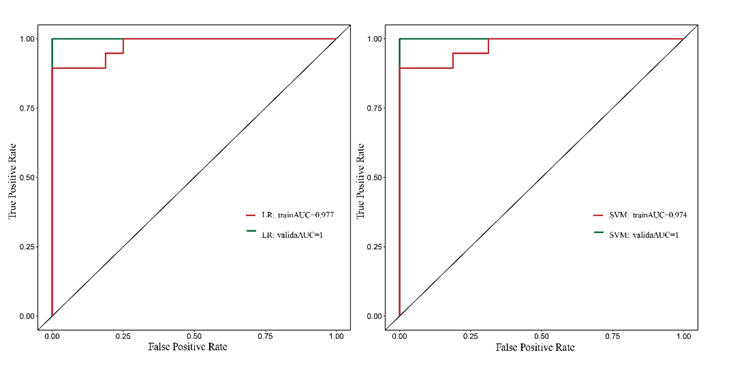


**Supplementary Figure 3.** ROC curves of other predict models. Green indicates the AUC of training set, red indicates the AUC of validation set. left: LR. right: SVM.
